# Heterogeneous and Allosteric Role of Surface Hydration for Protein–Ligand Binding

**DOI:** 10.1021/acs.jctc.2c00776

**Published:** 2023-02-23

**Authors:** Jie Shi, Jae-Hyun Cho, Wonmuk Hwang

**Affiliations:** † Department of Biomedical Engineering, Texas A&M University, College Station, Texas 777843, United States; ‡ Department of Biochemistry and Biophysics, Texas A&M University, College Station, Texas 77843, United States; ¶ Department of Biomedical Engineering, Texas A&M University, College Station, Texas 77843, United States; § Department of Materials Science and Engineering, Texas A&M University, College Station, Texas 77843, United States; ∥ Department of Physics and Astronomy, Texas A&M University, College Station, Texas 77843, United States

## Abstract

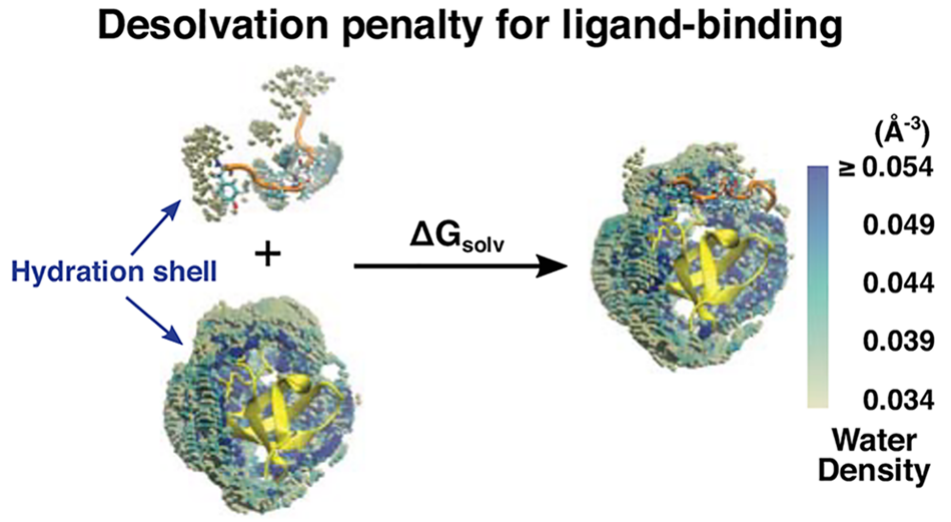

Atomistic-level understanding of surface hydration mediating protein–protein interactions and ligand binding has been a challenge due to the dynamic nature of water molecules near the surface. We develop a computational method to evaluate the solvation free energy based on the density map of the first hydration shell constructed from all-atom molecular dynamics simulation and use it to examine the binding of two intrinsically disordered ligands to their target protein domain. One ligand is from the human protein, and the other is from the 1918 Spanish flu virus. We find that the viral ligand incurs a 6.9 kcal/mol lower desolvation penalty upon binding to the target, which is consistent with its stronger binding affinity. The difference arises from the spatially fragmented and nonuniform water density profiles of the first hydration shell. In particular, residues that are distal from the ligand-binding site contribute to a varying extent to the desolvation penalty, among which the “entropy hotspot” residues contribute significantly. Thus, ligand binding alters hydration on remote sites in addition to affecting the binding interface. The nonlocal effect disappears when the conformational motion of the protein is suppressed. The present results elucidate the interplay between protein conformational dynamics and surface hydration. Our approach of measuring the solvation free energy based on the water density of the first hydration shell is tolerant of the conformational fluctuation of protein, and we expect it to be applicable to investigating a broad range of biomolecular interfaces.

## 1 Introduction

Surface water plays critical roles for the folding, conformational dynamics, and interaction between biomolecules,^1−5^ which arises from its behaviors that are distinct from those of bulk water. The first hydration shell tends to be higher in water density,^6−9^ and rotational and translational time scales of water molecules near protein surfaces are 3–7 times smaller than bulk values.^10,11^ Over 80% of known protein–ligand complexes have at least one water molecule at the binding interface,^12^ and there is an energy associated with displacing surface water upon binding of a ligand to a protein.^13^ Understanding of surface hydration in terms of the solvation pattern and its thermodynamic contribution to the binding free energy is an important consideration in ligand or drug design as well.^12,14−18^

Diverse experimental approaches, including X-ray crystallography,^6,19^ small-angle X-ray and neutron scattering,^8,20−22^ dielectric spectroscopy,^23,24^ ultrafast 2D-IR spectroscopy,^25−27^ and NMR spectroscopy,^28−35^ have been applied to elucidate how protein surface interacts with water molecules. Since these methods probe different aspects such as strongly bound water molecules or collective dynamics that are influenced by experimental conditions, molecular dynamics (MD) simulation has been instrumental for elucidating atomistic insight and for interpreting experimental data.^11,36,37^

Fluctuations of water can drive protein conformational motion.^38,39^ Conversely, proteins influence the behavior of the surrounding water via local surface topology, chemical composition, and conformational fluctuation.^3,40,41^ Surface hydration also plays a critical role for molecular recognition. Of interest is its contribution to the thermodynamics of ligand binding. Widely used methods such as free energy perturbation^42^ and thermodynamic integration^43^ calculate the total solvation contribution by decoupling the solvent from the solute gradually. The grand canonical Monte Carlo (GCMC) method has also been in wide use especially for finding hydration sites.^15,44^ However, these methods are mostly limited to a small number of bound water molecules.^45−47^ On the other hand, continuum solvation models employ the Poisson–Boltzmann equation and the nonpolar contribution via a simple scaling based on solvent accessibility of the solute surface.^48−51^ But continuum models do not account for features such as hydrogen bonds between water and the protein,^52^ water dipole reorientation, and water-mediated bridges, which limit their accuracy or applicability.^48,53,54^ It has been further suggested that for protein–protein complexes, continuum models often fail to reproduce experimental solvation free energy change as their nonpolar contributions are not simply linear in solvent-accessible surface area (SASA) and the Poisson–Boltzmann equation tends to underestimate the polar contribution.^55,56^ Inhomogeneous fluid solvation theory estimates the thermodynamics of water molecules through solute–solvent and solvent–solvent two-particle correlations.^57−59^ This method and its later developments have been applied to the solvation of small solutes or a small number of water molecules at hydration sites.^60−64^ However, the solute molecule has to be fixed so that the contribution of protein dynamics is not accounted for, and sampling issues arise for higher order correlations.^65^ Recently, a new approach was developed that calculates the rotational entropy of water using the K-nearest-neighbor density estimator combined with mutual information expansion.^65^ Yet, the method currently estimates only the rotational entropy of bulk water. There have also been detailed studies of hydration locally around the ligand binding pocket,^15,18^ yet sampling was either limited in the neighborhood of the ligand^15^ or it was based on many short, 35 ns MD simulations.^18^ While they may be well-suited for studying the binding of small compounds, effects of protein conformational motion on surface hydration are difficult to capture by these methods. Other approaches, though insightful, provide the entropy of the surface-bound water only,^66^ account for a limited number of water molecules around the site of interest, or require fixing the solute.^67−69^

While the diverse experimental and computational approaches explained above probe different aspects, accounting for the properties of hydration in a spatially resolved manner over the surface has been difficult. Here, we develop a computational approach to estimate the free energy of water molecules in the first hydration shell. It leverages the water density map constructed from MD simulation trajectory,^9,70^ so that the desolvation penalty upon binding of a ligand can be examined locally. To test our method, we used the N-terminal SH3 domain (nSH3) of a signaling adaptor protein CrkII and its two proline-rich motif (PRM) ligands, PRM^cAbl^ and PRM^NS1^. PRM^cAbl^ is from the C-terminal disordered region of the cAbl protein.^71,72^ PRM^NS1^ is from the C-terminal tail of the nonstructural protein 1 (NS1) of the 1918 Spanish influenza A virus that binds to nSH3 with a significantly higher affinity than PRM^cAbl^.^73,74^ The SH3 domains are one of the largest and best characterized modular binding domains found in more than 300 human proteins involved in various signaling processes,^75^ and PRMs binding to SH3 are one of the most abundant motifs mediating protein–protein interactions.^75,76^ Therefore, the SH3-PRM system forms an important model for protein–protein interactions. In particular, the nSH3-PRM complexes used in our study are well-characterized by X-ray, NMR, and binding kinetics measurements.^72−74^ Furthermore, we have previously characterized their backbone and side chain mobility changes upon ligand binding,^77^ which make them ideal for the present study.

We find a highly nonuniform distribution of the first hydration shell on the protein surface. Differences in the extent and water density of the shell in the unbound and ligand-bound states determine the desolvation penalty, which is less for the viral ligand PRM^NS1^. We also find a nonlocal effect where allosteric increase in the side chain motion in nSH3 distal from the ligand binding site reduces the desolvation penalty. Such an allosteric effect was absent in a control simulation where protein atoms were restrained as done in previous simulation studies analyzing surface hydration.^46,60,63,64,68,78^ Present results elucidate the importance of protein conformational dynamics in determining the heterogeneous and nonlocal nature of surface hydration, and our method of calculating the desolvation energy upon binding of a flexible ligand is applicable to quantitative analyses of molecular recognition in general.

## 2 Methods

### 2.1 Simulation Systems

We used the following systems:

1. nSH3:PRM^cAbl^: Protein Data Bank (PDB) code 5IH2 (1.8-Å resolution).^72^
2. nSH3:PRM^NS1^: PDB 6ATV (1.75 Å).^73^
3. nSH3: Isolated from nSH3:PRM^cAbl^.
4. PRM^cAbl^: Isolated from nSH3:PRM^cAbl^. Sequence: YEKPALPRKR.
5. PRM^NS1^: Isolated from nSH3:PRM^NS1B^. Sequence: YGRPPLPPKQKRK.
6. Bulk water without any protein.

All-atom MD simulations were performed using CHARMM^79,80^ with the param36 force field.^81^ For the first 5 systems, MD simulation trajectories from our previous work^77^ were used for the present analysis. Out of the 500 ns simulation time in each case, the 100–500 ns interval was used for analysis. The coordinate saving frequency was 5 ps. The bulk water simulation involved 1728 TIP3P^82^ water molecules in a cubic periodic box of side length of about 37.2 Å at 300 K. The production run was 10 ns long with a 0.5 ps coordinate saving frequency. The translational diffusion coefficient (*D*_*t*_) of water was calculated to be 0.61 Å^2^/ps, similar to the value for the TIP3P water model in previous studies.^9,83^ During Δ*t* = 0.5 ps, the molecule diffuses by  Å, which is greater than the 0.7-Å resolution for constructing the water density map explained below. Thus, our coordinate saving frequency for bulk water was large enough to avoid oversampling. In addition, to study the effect of suppressing protein motion on surface hydration, we performed 50 ns simulations of nSH3 and PRM separately or as a complex where protein atoms were positionally restrained with harmonic potentials of 50-kcal/(mol·Å^2^) spring constant.

### 2.2 Construction of Water Density Map

The water density map was constructed by adopting the method we developed previously.^9,70^ Briefly, the space around the protein is divided into cubic cells of side length 0.7 Å, which is about half the radius of a water molecule. The water density for each cell is the number of coordinate frames visited by a water oxygen atom divided by the product of the total number of frames used for analysis (80000 for solvated proteins and 20000 for bulk water simulation) and the volume of the cell (0.7 Å)^3^. The solvation map was constructed after aligning coordinate frames to a reference structure which was obtained by averaging the protein structure over the trajectory and performing a 300-step energy minimization in vacuum, to lightly relax atomic positions in the averaged structure. For aligning coordinate frames in simulations involving nSH3, the stably folded region in the unbound nSH3 was used where the root-mean-square-fluctuation (RMSF) of C_α_ atoms during the simulation was less than 0.5 Å. In simulations involving ligands only (PRM^cAbl^ and PRM^NS1^), since the RMSF of C_α_ atoms were above 1 Å, we used 4 C_α_ atoms in the central region that show relatively low RMSF in each system^77^ for alignment.

Water density maps were saved as the Medical Research Council (MRC) electron density map format files for visualization using UCSF Chimera.^84^ Maps were also saved as ASCII data files and used for calculating the desolvation energy. Both file formats are supported in CHARMM. A custom-written Python script was used to select cells of the water density map under different criteria and visualize using Visual Molecular Dynamics (VMD).^85^

### 2.3 Density-Based Solvation Free Energy Calculation

The water density map represents the preference of a water molecule to be in a given cell, which is an integral result of various factors including hydrogen bonding and local geometry of the protein surface near the cell. The free energy of water due to the nearby protein is thus given by
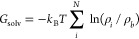
1where *k*_B_ is the Boltzmann constant, *T* is the temperature of the system, ρ_*i*_ is the water density of the *i*-th cubic cell, and *N* is the total number of cells within the first hydration shell selected via the procedure described below. ρ_*b*_ = 0.0333 Å^–3^ is the bulk water density where the zero point of the free energy is set. In this way, a higher surface water density contributes to lowering of *G*_solv_ (more negative). The desolvation energy (Δ*G*_solv_) upon binding of a ligand is calculated as the difference in *G*_solv_ between the complex and the protein and the ligand separately. A negative value of Δ*G*_solv_ indicates that the complex is preferred in terms of surface hydration.

To account for protein conformational motion, we calculate the water density of the *i*-th cell as follows
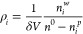
2where *δV* is the volume of the cubic cell, *n*_*i*_^*w*^ and *n*_*i*_^*p*^ are the number of frames that the cell *i* is visited by a water oxygen and by a protein atom, respectively, and *n*^0^ is the total number of frames. With the 0.7-Å cell size, it cannot be visited simultaneously by a water oxygen and a protein atom at any given frame, and away from the protein, *n*_*i*_^*p*^ = 0.

Since coordinate frames are oriented based on a reference structure, the simulation box takes a different orientation at each frame. This is due to the rotational motion of the protein, and it would occur even when the protein is kept near the center of the simulation box by imposing a center-of-mass constraint. As a result, a given cell near the boundary of a simulation box in a coordinate frame can lie outside of the simulation box in another frame that differs in orientation. Cells used for eq 1 should thus be away from the boundary of the simulation box to avoid undersampling. Ideally a fixed volume can be used that is smaller than the simulation box yet sufficiently larger than the protein (more than several times the thickness of the hydration shell). However, including cells that are too far away from the protein will make our calculation prone to error due to the density fluctuation in bulk water, while only a small fraction of cells is affected by the presence of the protein.

**Figure 1:**
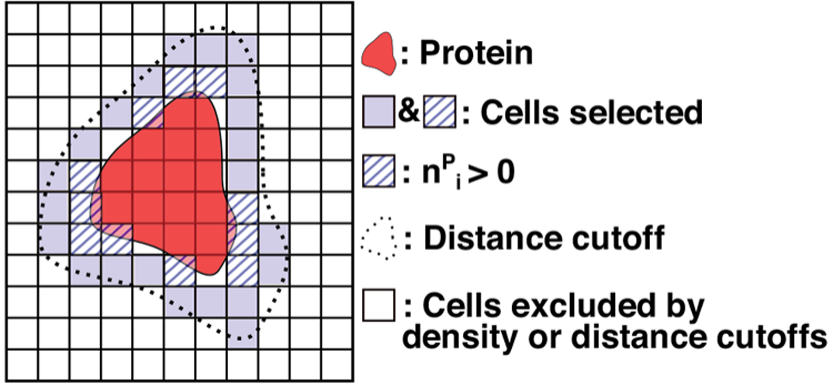
Criteria for selecting cells for solvation free energy calculation. A cell must be within a cutoff distance from the protein surface. The time-averaged water density of the cell is calculated based on coordinate frames where the cell is not visited by the protein. The area with a stripe pattern is selected cells with *n*_*i*_^*p*^ ≠ 0 in eq 2. If the density is below a water density cutoff, the cell is excluded from the summation in eq 1.

**Figure 2:**
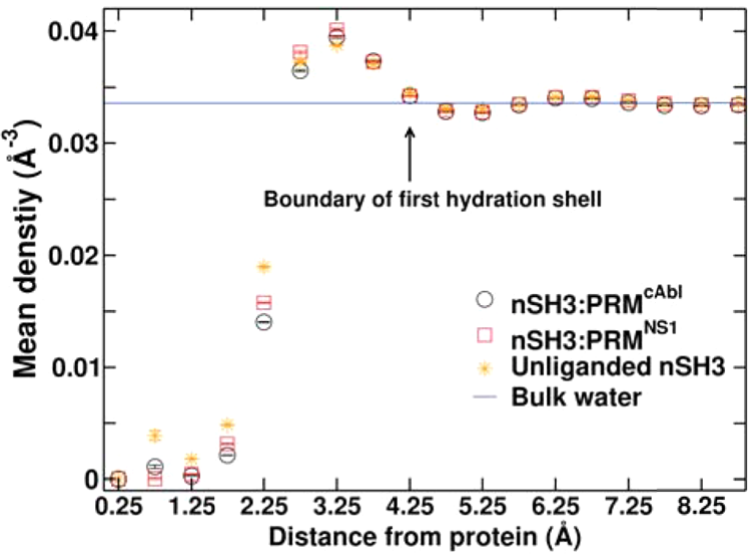
Average water density versus the distance from the protein. Distance was measured from the center of each cubic cell to the closest heavy atom of the protein structure used as the reference for aligning coordinate trajectories. Spatial averaging was carried out in 0.5-Å bins. The bulk water density (horizontal line at 0.0333 Å^–3^) was measured from a separate water-only simulation. The cutoff distance selected at 4.5 Å is marked by an arrow.

We thus introduce a distance cutoff to select cells belonging to the first hydration shell (Figure 1). Plots of the average water density versus the distance from the protein surface show density peaks corresponding to the first (3.25 Å) and the second (6.75 Å) hydration shells (Figure 2), similar to the spherically averaged radial distribution function.^8,59^ To select water molecules within the first hydration shell, we use a 4.5-Å cutoff that is slightly larger than the distance where the water density falls below the bulk value (vertical arrow in Figure 2). This is within the 4–8-Å range for the thickness of the first hydration shell estimated previously.^86^

**Figure 3:**
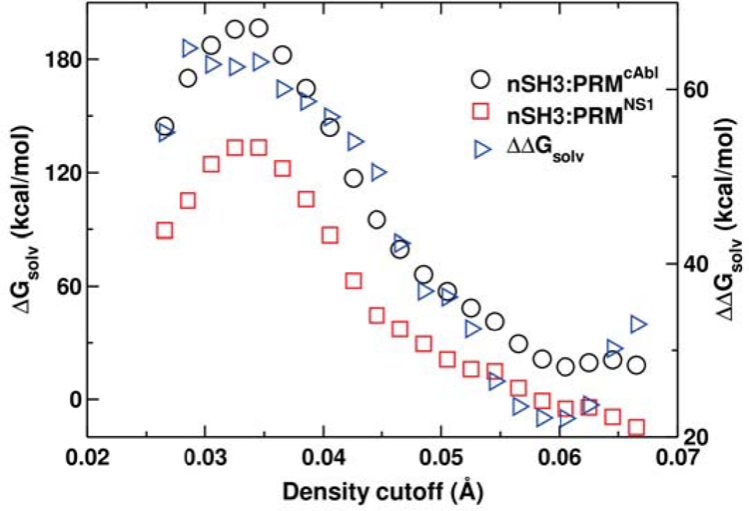
Dependence of the desolvation energy for ligand binding (Δ*G*_solv_) and the difference between the two systems (ΔΔ*G*_solv_) on the water density cutoff. ΔΔ*G*_solv_ > 0 indicates that the nSH3:PRM^cAbl^ complex experiences a greater desolvation penalty.

Water density tends to be higher near protein surfaces.^6,8,9,11^ On the other hand, certain cells near the protein may have a low water density due to steric exclusion. To exclude those cells, we varied the water density cutoff and measured Δ*G*_solv_ for nSH3:PRM^cAbl^ and nSH3:PRM^NS1^ (Figure 3). In both systems, Δ*G*_solv_ is maximal at 0.034 Å^–3^, which is slightly above the bulk value. The decrease in Δ*G*_solv_ at higher density cutoffs is because less cells are used for calculation, leading to fewer terms contributing to the summation in eq 1 (note that Δ*G*_solv_ is an extensive quantity). When the density cutoff becomes too high (reaching 0.058 Å^–3^), the desolvation energy for nSH3:PRM^NS1^ turns negative. This is because only a small number of cells (1958 in nSH3:PRM^NS1^, 1765 in unbound SH3, and 123 in unbound PRM^NS1^) have such a high water density. As explained in Results and Discussion, since the binding of PRM^NS1^ increases the local water density in some region near the nSH3 surface, more high-density cells become included in eq 1 for nSH3:PRM^NS1^ compared to the unbound case, which results in a negative desolvation energy. Conversely, cells with water density below the bulk value contribute positively to eq 1 (ρ_*i*_/ρ_*b*_ < 1), again leading to a reduction in Δ*G*_solv_. Based on these considerations, we used a 0.034-Å^–3^ density cutoff.

### 2.4 Free Energy Calculation Using the MM/GBSA Method

For comparison, the binding free energy between nSH3 and a PRM was calculated using the molecular mechanics/Generalized-Born (GB) surface area (MM/GBSA) method based on the following:^50,51^
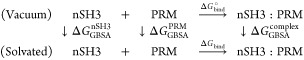


In the above, Δ*G*_bind_^°^ is the binding free energy in vacuum, given by
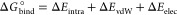
3where *E*_intra_ is the intramolecular energy associated with covalent bonds and bond angles, *E*_vdW_ is the van der Waals energy, and *E*_elec_ is the electrostatic energy in vacuum. These energy terms are calculated using the potential energy functions of the CHARMM param36 force field.^81^ The solvation free energy Δ*G*_GBSA_^*X*^ (*X*: PRM, nSH3, or complex) is
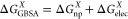
4where *G*_np_^*X*^ accounts for the nonpolar contribution proportional to SASA, and *G*_elec_^*X*^ is the free energy calculated using the GB with a simple switching (GBSW) module of CHARMM.^87^

The binding free energy Δ*G*_bind_ for the nSH3:PRM complex in solution is given by
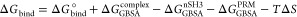
5

The conformational entropy *S* was calculated previously^77^ using the third-order maximum information spanning tree (MIST) method^88,89^ applied to the backbone and side-chain rotational angles. Entropy calculation for the uncomplexed PRM and nSH3 was based on simulations of respective monomers. Other energy terms for the uncomplexed monomers used trajectories of the complex where calculation was done separately for PRM and nSH3, as done previously.^50^ In this approach, Δ*E*_intra_ in eq 3 is zero; hence, it is not shown in Table 1.

**Table 1:** Decomposition of the Binding Free Energy^a^

	Δ*E*_vdW_	Δ*G*_np_	Δ*E*_elec_	Δ*G*_elec_	Δ*G*_sum_^GBSA^	–*T*Δ*S*	Δ*G*_solv_^*∞*^	Δ*G*_bind_	Δ*G*_bind_^′^
cAbl	–32.2 (6.6)	–4.4 (0.5)	–541.2 (162.6)	528.2 (156.6)	–49.6 (5.7)	8.6 (0.1)	27.3 (95.5)	–41.0 (5.8)	–13.8 (5.8)
NS1	–29.8 (6.2)	–4.9 (0.7)	–898.8 (117.9)	875.4 (115.3)	–58.2 (6.8)	11.7 (0.1)	20.4 (101.9)	–46.5 (6.9)	–26.1 (6.9)

a A negative value means that the energy is lower in the complex compared to the unbound state. First 4 terms: Energy terms in eqs 3-4. Δ*G*_sum_^GBSA^: the sum of the first 4 terms. −*T*Δ*S*: entropy contribution at *T* = 300 K. Δ*G*_solv_^*∞*^: water density-based desolvation energy (Figure 5). Δ*G*_bind_ = Δ*G*_sum_^GBSA^ – *T*Δ*S* (eq 5). Δ*G*_bind_^′^= Δ*G*_bind_ + Δ*G*_solv_^*∞*^. Uncertainties (in parentheses) for the first 5 terms are standard deviations across the coordinate frames. Uncertainty in entropy is from ref (77). Uncertainties for the last 3 columns are propagated values.

## 3 Results and Discussion

### 3.1 Selecting the First Hydration Shell

**Figure 4:**
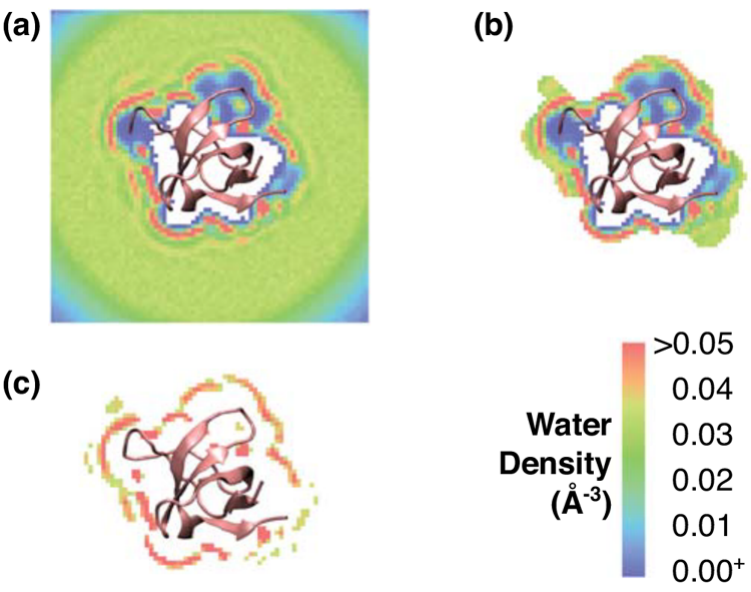
Selecting cells for desolvation energy calculation. For illustration, a single 0.7-Å thick slice of the water density map for the unbound nSH3 is shown, which makes parts of the water density map appear to be truncated. Excluded cells as well as cells with zero water density are not colored (white). (a) No cutoff applied. Up to 4 hydration shells can be seen (density in red). (b) 4.5-Å distance cutoff applied (Figure 2). (c) Both 4.5-Å distance cutoff and 0.034/Å^3^ density cutoff (Figure 3) applied.

To exclude bulk water in the simulation system when calculating the desolvation energy, surface water needs to be identified, for which we impose distance and water density cutoffs (Figure 1; see Methods). To see the effects of imposing cutoffs, we first construct a water density map without any cutoff applied (Figure 4a). The hollow region inside the protein corresponds to cells where water molecules are sterically occluded (white in Figure 4a). The low water density in the corners of the box (blue in Figure 4a) is due to undersampling caused by reorienting the original simulation box to the reference structure frame-by-frame (see Methods). Near the protein surface, up to four hydration shells are visible (red stripes in Figure 4a). Due to their discontinuity, hydration shells are less pronounced when averaged over distance (Figure 2). We keep only the first hydration shell by imposing a 4.5-Å distance cutoff (Figure 4b).

To further exclude bulk water and the region near the protein that have low water density due to steric effect, we also apply a 0.034/Å^3^ density cutoff that is slightly higher than the bulk value (Figure 4c). The selected cells are used for desolvation energy calculation. Because of the density cutoff, accounting for the frames visited by a protein atom for a selected cell becomes negligible, where *n*_*i*_^*p*^ in eq 2 is less than 0.3% of *n*^0^.

### 3.2 Density Map-Based Desolvation Energy

**Figure 5:**
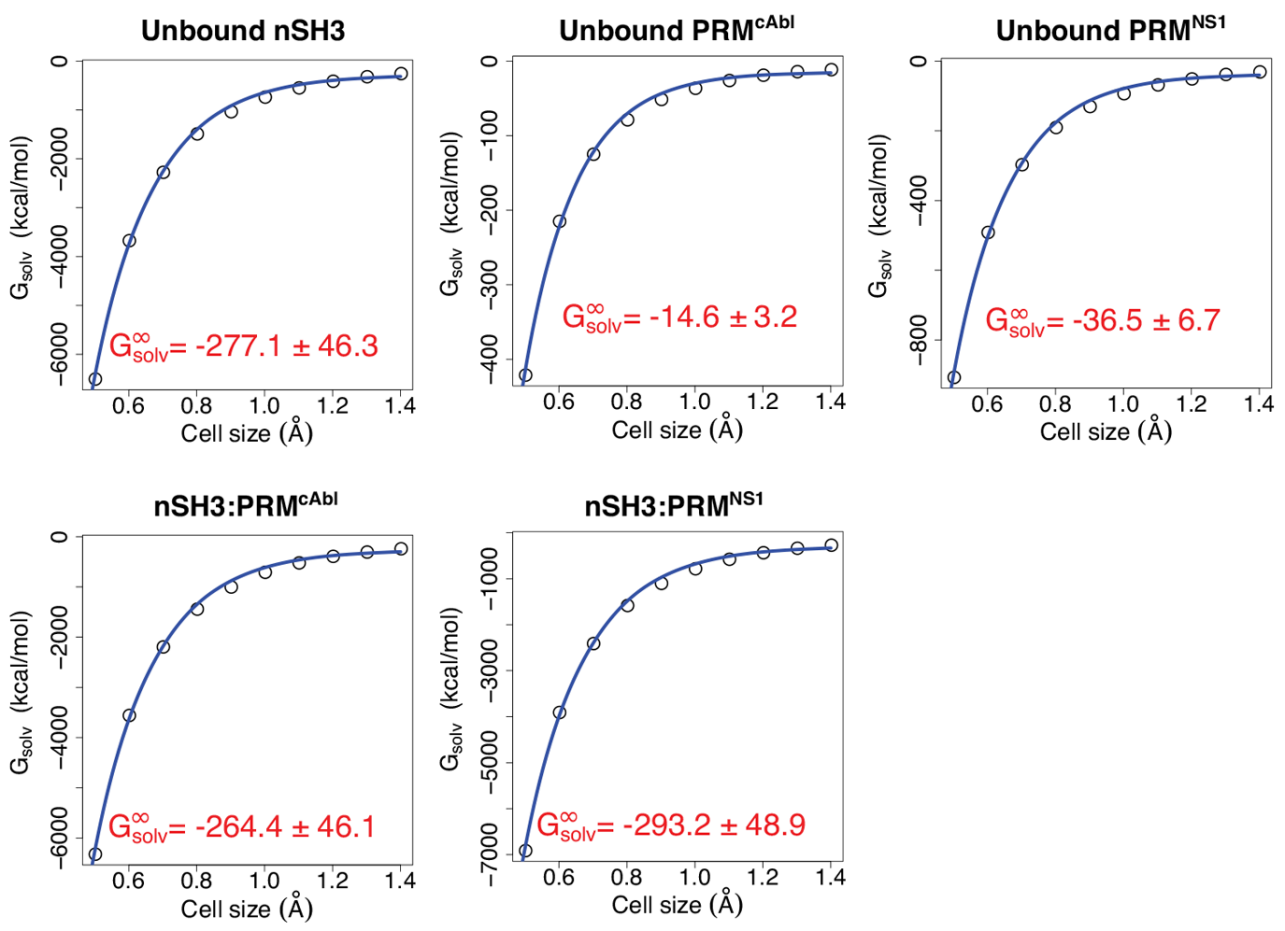
Dependence of *G*_solv_ on the spatial resolution of the water density map. Open circle: Calculated *G*_solv_. Blue line: Fit with *G*_solv_ = *a* × *e*^–*bx*^ + *G*_solv_^*∞*^ (*x*: cell size). The asymptotic value of the fit is noted in each panel. The uncertainty is based on nonlinear regression.^90^

The water density map used for eq 1 depends on the size of the cell. A smaller cell will increase the spatial resolution of the map but at the cost of increased noise due to low statistics for each cell. A converse effect applies when using a larger cell. Our initial choice was 0.7 Å, which is about half the radius of a water molecule. To examine the dependence of *G*_solv_ on the cell size, we varied the latter between 0.5 and 1.4 Å (Figure 5). As a larger cell size improves sampling, we fitted *G*_solv_ versus the cell size with an empirical equation (Figure 5) and used the asymptotic value for comparing the desolvation energy upon complex formation (Δ*G*_solv_^*∞*^ in Table 1). Comparing between nSH3:PRM^cAbl^ and nSH3:PRM^NS1^, the latter is lower in Δ*G*_solv_^*∞*^ by 6.9 kcal/mol, suggesting that the viral ligand PRM^NS1^ incurs less desolvation penalty when binding to nSH3, which is consistent with its stronger binding.^72,73^ The lower desolvation penalty of nSH3:PRM^NS1^ is observed in nearly all of the water density cutoffs tested except with very high cutoffs where statistics is less reliable (Figure 3), which supports the robustness of the above result regarding the two PRMs.

### 3.3 Contribution of Surface Hydration to the Binding Free Energy

We assessed the free energy of ligand binding in the two systems studied via the MM/GBSA method^48,91^ and also compared it with the desolvation penalty calculated using our method. First, our MM/GBSA results are consistent with previous studies (Table 1)^50,51,92,93^ in that electrostatic terms (Δ*E*_elec_ and Δ*G*_elec_) are much larger in magnitude than the van der Waals and nonpolar terms, but they mostly cancel each other when added, so that the sum of the van der Waals and nonpolar terms (Δ*E*_vdW_ + Δ*G*_np_) plays a greater role. Also, whereas Δ*G*_bind_ values in Table 1 are significantly lower (larger in magnitude) than experimental values (−7.63 kcal/mol for cAbl and −11.36 kcal/mol for NS1),^72,73^ the 5.48-kcal/mol difference between PRM^cAbl^ and PRM^NS1^ is close to the experimental 3.73 kcal/mol, again consistent with previous studies.^93,94^

The conformational entropy was calculated using the MIST method.^77^ A previous computational study on the binding of 15 different PRMs to an SH3 domain used normal-mode analysis to calculate the conformational entropy, which did not find any clear difference among the binder PRMs.^93^ Since normal-mode analysis relies on the structure at a potential energy minimum, it cannot account for amino acid side-chain rotation over an energy barrier, which plays an important role for the conformational entropy.^95,96^ MIST uses the side chain and backbone dihedral angles measured in simulation trajectories as order parameters so that transitions among energy minima are accounted for. As we noted previously,^77^ nSH3:PRM^NS1^ experiences greater entropy loss upon complex formation (3.1 kcal/mol higher – *T*Δ*S* than nSH3:PRM^cAbl^; Table 1), which offsets the larger difference in Δ*G*_sum_^GBSA^. In comparison, the desolvation penalty Δ*G*_solv_^*∞*^ is smaller for nSH3:PRM^NS1^, indicating that the viral ligand keeps surface hydration more bulk-like when binding to nSH3, which is consistent with the higher affinity of the viral ligand.

In the MM/GBSA approach, the solvation effect is incorporated by the polar and nonpolar terms (eq 4).^48,97^ The polar term relates to the electrostatic screening, which does not consider the presence of the hydration shell. The nonpolar term is distantly related to our water density-based desolvation energy since ligand binding leads to changes in both SASA and surface hydration. However, Δ*G*_solv_^*∞*^ is 4 to 6 times larger in magnitude than Δ*G*_np_ (Table 1), indicating that changes in surface hydration play a greater energetic role than surface tension-based energy changes associated with SASA, which is in line with previous studies.^55,56^ Since Δ*G*_solv_^*∞*^ is not captured by the continuum-based MM/GBSA calculation, we add them to obtain the net free energy of binding Δ*G*_bind_^′^ (Table 1).

### 3.4 Changes in Surface Hydration upon Ligand Binding

To elucidate the structural origin for the lower desolvation penalty of PRM^NS1^, we counted the number of cells included in eq 1 for the unbound and bound systems. Since the 0.034-Å^–3^ density cutoff for selecting cells is above the bulk water density, a greater number of selected cells in the bound state contribute to decreasing Δ*G*_solv_, hence more favorable hydration. For the unbound state, let the number of cells around the ligand and nSH3 be *N*_*u*_^lig^ (lig: cAbl or NS1) and *N*_*u*_^nSH3^, respectively, and let the number of cells in the bound state be *N*_*b*_^lig+nSH3^. The difference  is −1600 for PRM^cAbl^ and −1940 for PRM^NS1^. The negativity of values indicates that binding of a ligand reduces the number of cells comprising the first hydration shell, as expected from burial of the interfacial area. This effect is greater for PRM^NS1^ because it is 3-aa longer than PRM^cAbl^.

**Figure 6:**
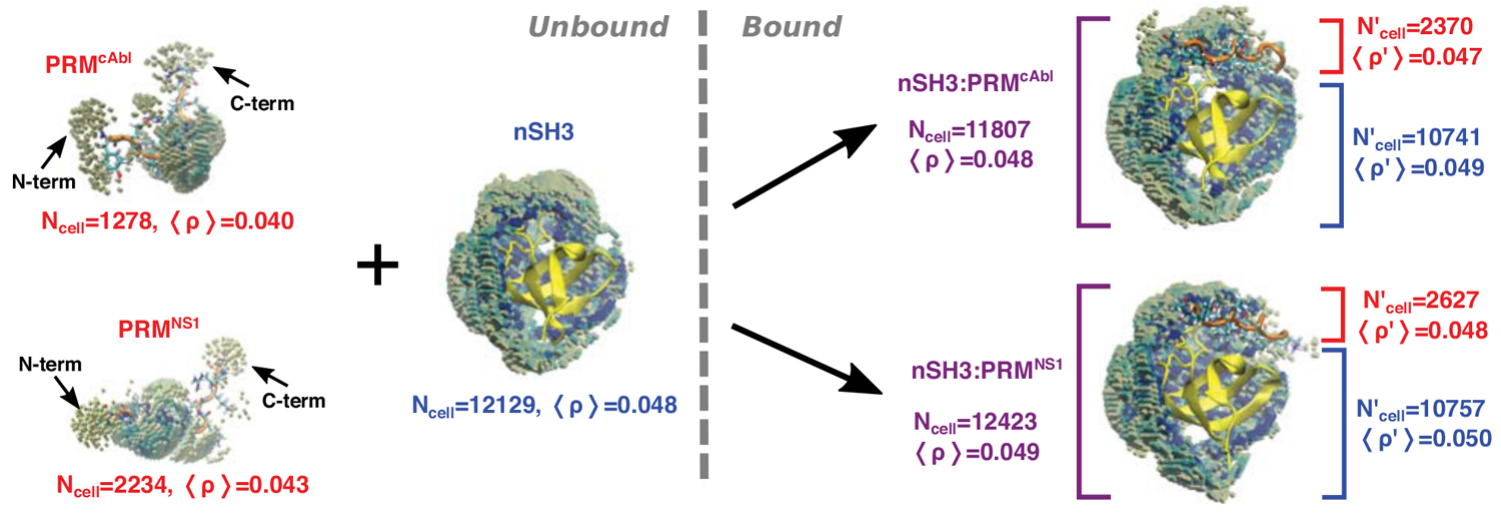
Changes in surface hydration upon complex formation. *N*_cell_: number of cells selected for eq 1. ⟨ρ⟩: mean water density in the selected cells. N-term/C-term denote the termini of ligands. *N*_cell_^′^ and ⟨ρ′⟩: values calculated separately for the ligand and nSH3 in the complex. Cells in front of nSH3 are partially removed to show the structure.

For a ligand-bound system, we separately counted the number of cells within 4.5 Å from the ligand and from nSH3, respectively (Figure 6, *N*_cell_^′^). Comparison with the corresponding numbers in unbound cases informs respective changes in surface hydration. For nSH3, the loss of hydration was comparable between binding of the two ligands (Figure 6, 10741 vs 10757, compared to 12129 for the isolated nSH3). On the other hand, both ligands have more cells with high water density when bound to nSH3, where PRM^cAbl^ gains 699 more cells than PRM^NS1^ (1278 → 2370 for PRM^cAbl^ vs 2234 → 2627 for PRM^NS1^). The increase is because the ligands are intrinsically disordered in the unbound state, so that the surrounding water is more bulk-like, while the extended conformation when bound to nSH3^98^ leads to the formation of a more stable hydration shell. The increase in the stable hydration shell due to reduction in conformational motion is large enough to compensate for the loss of SASA of the ligand upon binding to nSH3.

PRM^cAbl^ gains about twice more cells when bound to nSH3. This is because of the much smaller *N*_cell_ (1278) compared to that in PRM^NS1^ (2234) in the unbound state, while *N*_cell_^′^ varies less in the bound states (Figure 6, 2370 vs 2627). In an unbound PRM^cAbl^, E759 on the N-terminal side forms dynamic salt bridges with the 3 positively charged residues on the C-terminal side, which makes the peptide bent on average (Figure 6, top left). Possessing no negatively charged residues, PRM^NS1^ stays more extended, hence resulting in a smaller difference in the extent of hydration between the bound and unbound states.

We measured SASA that is commonly used for calculating the nonpolar energy (Δ*G*_np_ in eq 4).^87,99^ For unbound PRM^cAbl^ and PRM^NS1^, it is 1765 ± 151 Å^2^ and 2239 ± 122 Å^2^, respectively (mean ± sd). When bound to nSH3, it is 1327 ± 69 Å^2^ (PRM^cAbl^) and 1673 ± 67 Å^2^ (PRM^NS1^). The comparatively smaller SASA of the unbound PRM^cAbl^ reflects the bent conformation mentioned above, and the larger fluctuation (151 Å^2^ for PRM^cAbl^ vs 122 Å^2^ for PRM^NS1^) is due to the transition between the bent and extended states, which reflects greater difficulty in forming a stable hydration shell. This is consistent with the smaller *N*_cell_ for the unbound PRM^cAbl^.

If the density of water in selected cells were identical, having a smaller reduction in the number of cells  for PRM^cAbl^ would indicate a less desolvation penalty. However, the density distribution is nonuniform, and it is higher in cells selected for nSH3:PRM^NS1^, which result in a lower desolvation penalty (Δ*G*_solv_^*∞*^ in Table 1). As a test, we measured the average water density ⟨ρ⟩ in each case and estimated the free energy of the hydration shell in each case by using −*N*_cell_*k*_B_*T* ln(⟨ρ⟩/ρ_*b*_). The resulting desolvation penalty of ligand binding is 229.6 kcal/mol (nSH3:PRM^cAbl^) and 163.5 kcal/mol (nSH3:PRM^NS1^), which are comparable to the values based on eq 1 (Table S1, 201.6 kcal/mol (cAbl) and 159.3 kcal/mol (NS1) at 0.7 Å). Upon binding to nSH3, PRM^NS1^ incurs a larger decrease in side-chain entropy,^77^ which contributes to increasing the density of surface water.

### 3.5 Allosteric Role of the Side-Chain Motion for Desolvation Energy

**Figure 7:**
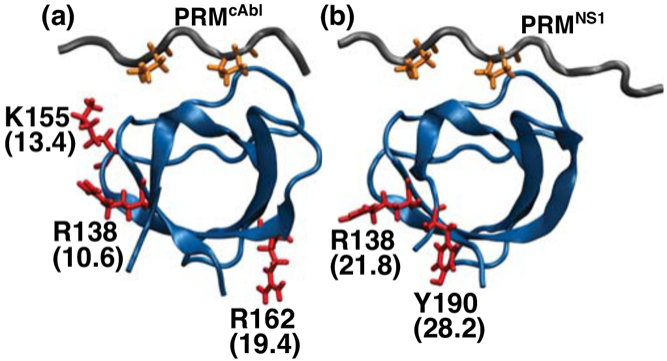
Entropy hotspot residues contributing to a large desolvation penalty upon ligand binding. Δ*G*_solv_ calculated for each residue is in parentheses, in kcal/mol. See Tables S2 and S3. (a) nSH3:PRM^cAbl^ and (b) nSH3:PRM^NS1^. Orange sticks: two prolines in the central proline-rich motif for each ligand, shown as a visual guide.

We analyzed residue-level changes in surface hydration by applying the same criteria for selecting cells as explained above to heavy atoms of individual residues of nSH3 (Tables S2 and S3). Overall, residues at the ligand binding site, defined as those that have nonzero contact occupancy with the ligand during the simulation, contribute more to the desolvation penalty, with values in the range of −13.3 to +51.6 kcal/mol for nSH3:PRM^cAbl^ (Δ*G*_solv_ in Table S2) and −19.5 to +54.4 kcal/mol for nSH3:PRM^NS1^ (Table S3). However, residues of nSH3 that do not contact the ligand also contribute substantially, ranging from −14.3 to +19.4 kcal/mol (nSH3:PRM^cAbl^) and −53.8 to +28.2 kcal/mol (nSH3:PRM^NS1^). Among remote residues that incur the greatest desolvation penalty (the largest positive Δ*G*_solv_) are “entropy hotspots” that allosterically increase their side-chain entropy upon ligand binding (Figure 7).^77^ Upon ligand binding, their greater side-chain mobility leads to disruption of the surrounding hydration shell, thereby resulting in greater desolvation penalty.

We also examined hydration around residues that lose side-chain entropy upon ligand binding,^77^ which potentially have a reverse effect. For nSH3:PRM^cAbl^, those residues are located only in the ligand-binding interface. For nSH3:PRM^NS1^, K178 loses entropy the most among distal residues (0.76 kcal/mol at 300 K),^77^ but it involves only a moderate decrease in Δ*G*_solv_ (−5.1 kcal/mol; Table S3). The residue has a large number of surrounding cells (*N*_cell_; Table S3). Since it is already well-hydrated, reduction in side-chain entropy upon ligand binding does not cause any further increase in surface hydration.

There are a few other residues that are not entropy hotspots but involve comparable Δ*G*_solv_. Also, certain remote residues have large negative Δ*G*_solv_ (Tables S2 and S3), suggesting that they become more stably hydrated upon ligand binding. Given the complex interplay of contacts between flanking residues on the surface that affect local conformation and solvent accessibility,^77^ it is difficult to pinpoint a clear mechanism for their solvation energy change. Taken together, all side-chain entropy hotspots increase the desolvation penalty of ligand binding, but the converse does not hold–not all residues that contribute substantially to the desolvation penalty are entropy hotspots. The present results also demonstrate that the hydration state of remote residues can be significantly affected by ligand binding.

### 3.6 Dependence of Desolvation Energy on the Choice of Orientational Reference

**Figure 8:**
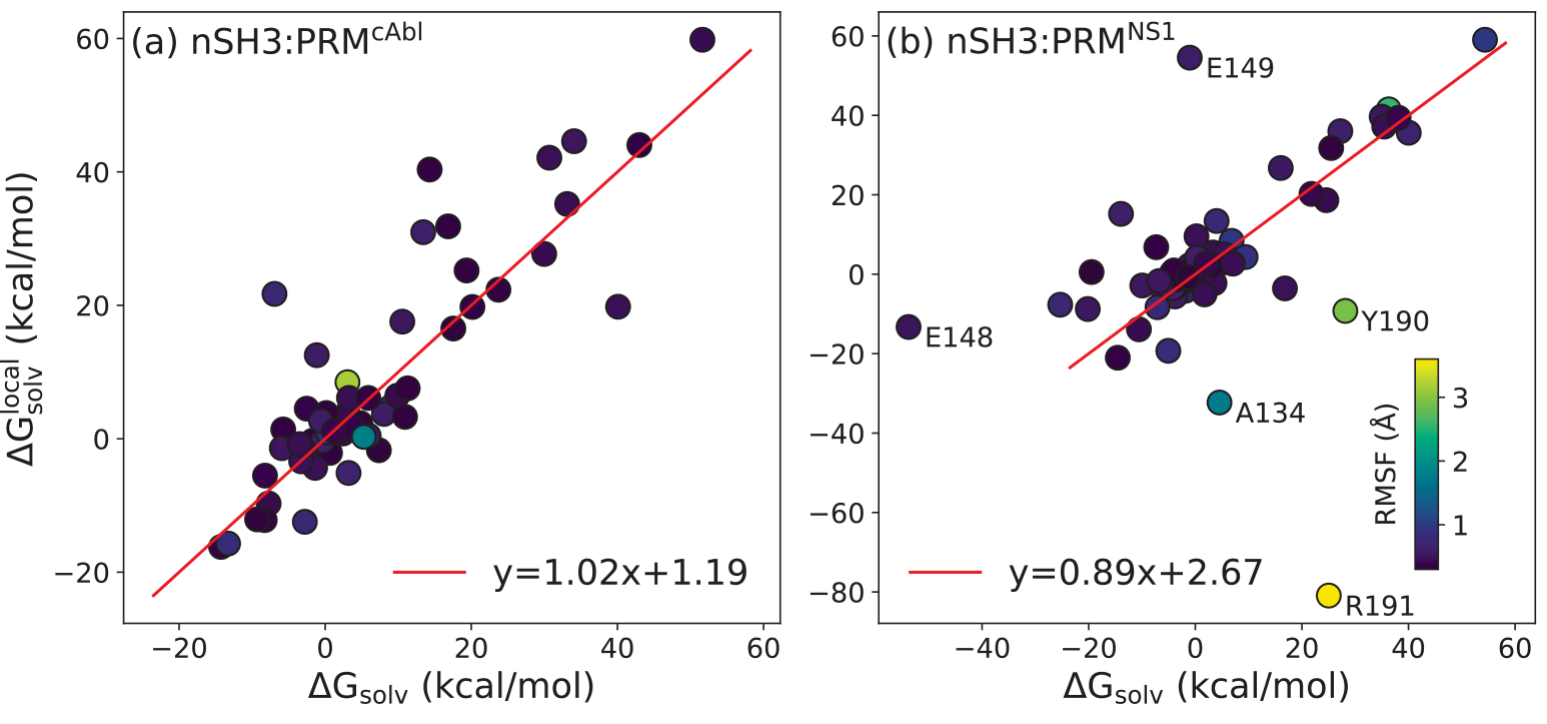
Effect of the orientational reference on desolvation penalty calculation. Δ*G*_solv_: Desolvation penalty for each residue in our original calculation where low-RMSF C_α_ atoms of nSH3 were used as orientational reference. Δ*G*_solv_^local^: Case when the backbone and C_β_ atoms of each residue were used as orientational reference. (a) nSH3:PRM^cAbl^, (b) nSH3:PRM^NS1^. Each dot is colored according to the RMSF of the corresponding C_α_ atom.^77^ In (b), high-RMSF residues at the N- and C-terminal ends of nSH3 (A134, Y190, and R191), E148, and E149 that exhibit large differences between Δ*G*_solv_ and Δ*G*_solv_^local^ are labeled. Red lines are linear fits. In (b), the 5 labeled residues were excluded from the linear fit. Individual values are in Tables S2 and S3.

Our water density map calculation relies on aligning coordinate frames based on reference atoms, for which we used C_α_ atoms of nSH3 with lower than 0.5-Å RMSF (Methods). A potential issue with this method is that the calculated water density becomes low around a flexible region in contrast to the case when coordinate frames are aligned more locally around the region, as the hydration water may move together with it.^100^ For regions that remain flexible before and after ligand binding, using the whole nSH3 as reference is unlikely to cause any serious error since water density around those regions would be similar and low, so that they are excluded from our calculation with the 0.034/Å^3^ density cutoff. In other words, the hydration state of the flexible region does not change so that it is not considered for desolvation energy calculation. However, there may be regions that change flexibility or conformation upon ligand binding, which can impact the desolvation penalty calculation. To investigate this possibility, we aligned coordinate frames locally for each residue using its backbone atoms and the C_β_ atom as the orientational reference and calculated the desolvation penalty denoted by Δ*G*_solv_^local^ (Figure 8; Tables S2 and S3). Linear fits (excluding 5 residues in nSH3:PRM^NS1^; explained below) have a slope close to 1 with small values of *y*-intercept (red lines in Figure 8), indicating that using the whole nSH3 domain (global) or individual residues (local) as orientational reference yield consistent results in desolvation penalty calculation.

Compared to nSH3:PRM^cAbl^, nSH3:PRM^NS1^ involving the viral ligand had more mismatches in the two calculations (labeled in Figure 8b). In particular, R191 at the C-terminus of nSH3 had the largest discrepancy. Its C_α_ RMSF remains similar between unliganded nSH3 (2.1 Å) and when PRM^cAbl^ binds (1.8 Å), while it increases to 3.6 Å when PRM^NS1^ binds, which is the largest RMSF value of all residues of nSH3 in the two systems. When the whole nSH3 is used as the orientational reference, the hydration shell around R191 thus becomes less defined when a PRM^NS1^ binds, leading to Δ*G*_solv_ > 0. But when R191 is used as the orientational reference, its large fluctuation makes the positional relation between its side chain and the rest of the protein difficult to predict, which may have resulted in a large negative value of Δ*G*_solv_^local^. A134 at the N-terminus of nSH3 flanks R191, both located at the ends of terminal β-strands, which may thus have been influenced by the increased motion of R191 in nSH3:PRM^NS1^. A similar situation occurs for E148 and E149. E149 is at the ligand-binding pocket (Table S3), and it has a large positive Δ*G*_solv_^local^ compared to Δ*G*_solv_ in nSH3:PRM^NS1^ (Figure 8b). Likely affected by this, its neighboring residue E148 also shows a substantial increase in Δ*G*_solv_^local^ compared to Δ*G*_solv_, though it still remains negative (Figure 8b).

Although Δ*G*_solv_ and Δ*G*_solv_^local^ calculated for each residue cannot be added to yield the desolvation penalty of ligand binding as a whole, the above comparison shows that the heterogeneity and nonlocality in the contribution of residues to the desolvation penalty do not depend sensitively on the choice of the reference for aligning coordinate frames. This is because nSH3 is a well-folded protein with overall low RMSF. Also, mismatches between Δ*G*_solv_ and Δ*G*_solv_^local^ may be used to identify residues that change solvation behavior upon ligand binding, either around the binding pocket or away from it.

### 3.7 Significance of Protein Motion for Surface Hydration

In previous computational approaches to calculate the solvation free energy, protein atoms were positionally restrained, and only water was allowed to move.^13,58,63^ This would be suitable for studying stable hydration sites or ordered water molecules inside relatively immobile binding pockets. However, restraining proteins alters the surface hydration in mobile loops or around amino acid side chains that have high mobility. As a test, we performed a separate simulation with a 50-kcal/mol·Å^2^ harmonic restraint applied to protein atoms. Since only the picosecond-scale motion of water molecules needs to be sampled,^11^ we performed the simulation for 50 ns.

**Figure 9:**
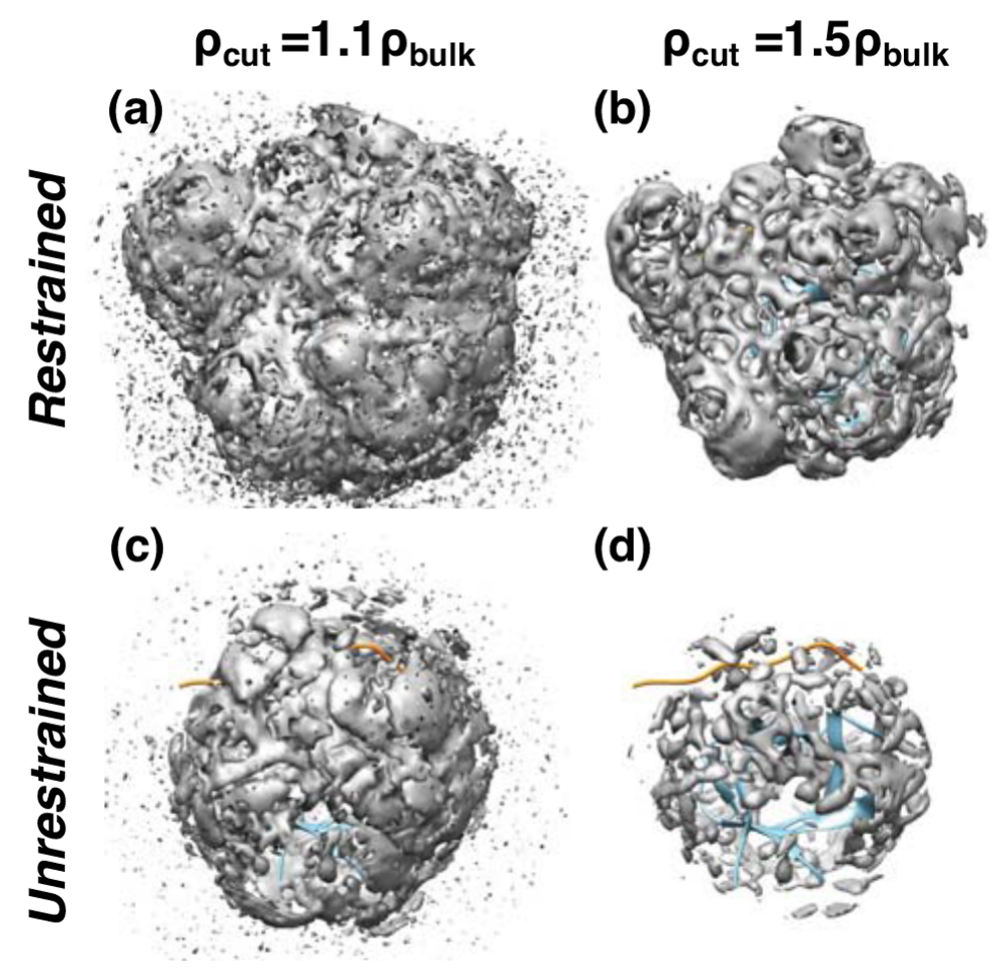
Testing the influence of protein conformational motion on surface hydration. The hydration map of nSH3:PRM^cAbl^ is shown as an example. (a,b) Protein atoms positionally restrained with a 50-kcal/mol·Å^2^ harmonic potential throughout the simulation. (c,d) Simulation with no restraint. The hydration map was visualized with two different density cutoffs, as indicated on top (ρ_*b*_ = 0.0333 Å^–3^: bulk water density).

In all systems, the high-density region covers the protein much more extensively compared to the case without any restraint (Figure 9). The mean water density within the 4.5-Å distance from the protein (0.064–0.077 Å^–3^; Table 2) is about 1.7 times greater than the unrestrained case (0.044 Å^–3^), where the latter is close to the experimental estimate, 0.04 Å^–3^.^6,8^ The greater number of cells and higher water density make *G*_solv_^*∞*^ also significantly larger in magnitude (more negative; Figure 5 and Table S1 vs Table 2). The positional restraint has greater influence on the unbound ligands that are intrinsically disordered. As a result, the desolvation penalty of ligand binding (Δ*G*_solv_) increases by 5.4-fold (cAbl) to 6.6-fold (NS1). Furthermore, residue-level analysis of surface hydration (Tables S4 and S5) shows that the desolvation penalty of remote residues decreases substantially in magnitude and, conversely, it increases for residues at the ligand-binding interface, making the relative contribution of the latter much greater than the case without any restraint applied, which indicates that protein conformational motion is necessary for the allosteric effect of ligand binding on surface hydration.

**Table 2:** Effect of Restraining Protein Atoms on Hydration^a^

	*N*_cell_	⟨ρ⟩ (Å^–3^)	*G*_solv_^*∞*^ (kcal/mol)	Δ*G*_solv_^*∞*^ (kcal/mol)
nSH3 (cAbl)	11573 (32)	0.076	–567.3 (88.1)	
nSH3 (NS1)	11301 (23)	0.077	–551.1 (84.3)	
PRM^cAbl^	5932 (14)	0.064	–232.2 (37.8)	
PRM^NS1^	6672 (20)	0.064	–262.2 (40.8)	
nSH3:PRM^cAbl^	13869 (21)	0.074	–653.1 (100.1)	146.4 (226.0)
nSH3:PRM^NS1^	14059 (16)	0.076	–678.5 (106.4)	134.8 (231.5)

a Calculation is based on a 50-ns simulation with a 50-kcal/mol·Å^2^ harmonic positional restraint applied to each protein atom throughout the simulation. Due to the restraint, conformations of nSH3 differ between the complexes with PRM^cAbl^ and PRM^NS1^, so two separate simulations were done for unbound nSH3 in respective cases. ⟨ρ⟩: Mean water density in selected cells (*cf.*, Figure 6). Its standard deviation, as measured by subsampling the 50-ns trajectories every eighth frame (a total of eight sets), is less than 1% of ⟨ρ⟩. Uncertainties in energies (in parentheses) are measured in the same way as in Table 1.

### 3.8 Comparison with Grid Inhomogeneous Solvation Theory

The inhomogeneous fluid solvation theory^57−59,64^ and its discretized version, grid inhomogeneous solvation theory (GIST),^62,63,101^ have been widely used for solvation free energy calculation. GIST utilizes a setup similar to our approach, where a region of interest is divided into a grid and water properties are calculated for each cell (also called voxel). As noted above, currently this approach relies on simulations with protein or solute atoms positionally restrained, which results in uniform and dense hydration as in Figure 9a,b. While direct comparison between our calculation without restraint and GIST is difficult, it is instructive to discuss about the measured quantities in the two methods. In our approach, we use the local water density as a measure of free energy. As water molecules sample spaces, a higher water density (frequency of visitation) indicates that the cell is a preferred spot, which is a result of various factors such as the interaction of the water in the cell with nearby protein atoms or water molecules. In turn, water molecules influence protein motion, so that a high-density cell is formed through an interplay between protein and water. In comparison, GIST calculates the interaction energy for water molecules directly from the force field used in the simulation, and the entropy of water from radial and orientational pair correlation functions with the surrounding protein and water.^62,101^ In GIST, water density is considered to be due to a “packing effect,” rather than playing any energetic role.^62^ Indeed, when protein atoms are restrained, the protein surface becomes a static boundary, so that a higher water density is largely due to the “hard-wall effect”.^102,103^ In contrast, a mobile protein surface presents a moving boundary so that the density of the hydration shell is diminished, as shown in our simulations with and without restraints (Figure 9).

**Figure 10:**
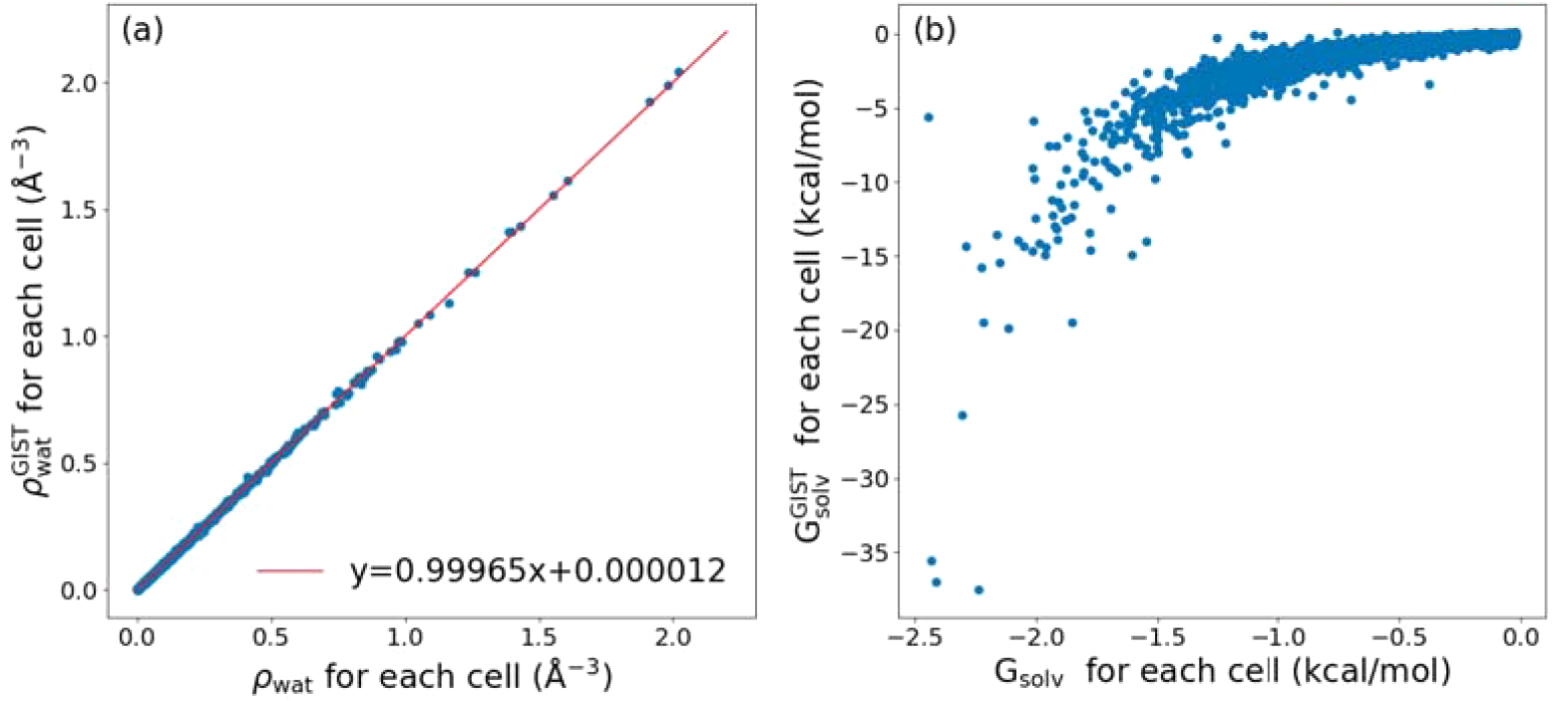
Cell-by-cell comparison with GIST. Calculation is based on the 50-ns simulation trajectory of unliganded nSH3 with protein atoms restrained, as in Figure 9a,b. (a) Water density and (b) solvation energy for each cell. *x*-axis: Values are based on our method. *y*-axis: Values are based on GIST. In (a), all cells with nonzero water density are used. Red line: linear fit. In (b), only cells in the first hydration shell selected by our methods are used. The GIST calculation was performed by using CPPTRAJ.^104^

To compare the solvation energy calculation by our approach and by GIST, we applied the two methods to the simulation trajectory of an unliganded nSH3 with protein atoms restrained. The water densities calculated by using the two methods for each cell were nearly identical, where slight discrepancy is likely due to roundoff errors in assigning individual water oxygen atoms to cells (Figure 10a). For each cell in the first hydration shell as selected by our criteria, we calculated the individual term on the right-hand side of eq 1 and also calculated the solvation energy by using GIST (Figure 10b). There is a positive correlation between the two values, which confirms that our density-based solvation free energy does capture the effect of the interaction energy and entropy of water in GIST. However, the GIST energy varies over a range nearly 16-fold greater than our *G*_solv_ (Figure 10b, about 40-kcal/mol vs 2.5-kcal/mol range). Examination of the GIST energy terms shows that large negative solvation energies (*G*_solv_^GIST^ less than −25 kcal/mol in Figure 10b) are mostly due to the water-solute interaction energy (−53.9 to −28.3 kcal/mol), followed by the entropic contribution (−12.3 to −7.3 kcal/mol). These cells are high in water density and close to proteins. Their close proximity to protein atoms may have resulted in a large negative interaction energy. Entropy depends on the logarithm of the pairwise correlation function, which varies rapidly in the interval between 0 and 1. In comparison, the terms on the right-hand side of eq 1 consist of logarithm of a number greater than 1, which varies more slowly. Considering that the thermal energy at 300 K is 0.6 kcal/mol, the large range of free energies of water across different cells in GIST appears to be less realistic compared to that of our method.

In principle, it is possible to calculate GIST-like energy and entropy terms in simulation trajectories without any positional restraint applied to protein atoms. Apart from the difficulty of dealing with protein motion, sampling becomes a practical challenge for such calculation. In particular, in order to calculate the pair correlation function for estimating the entropy of water, a large number of frames are needed so that a select set of frames can be further subdivided into bins for the correlation function with enough counts for each bin. While using a short time interval between saving coordinate frames will increase the sample size, if it is shorter than the time scale of water motion, oversampling may ensue. Coordinate saving frequency in some of previous GIST calculations ranges from 0.36 to 2 ps,^62−64,101^ which are comparable to or less than the lifetime of water–protein hydrogen bond or water tumbling time. While those intervals may be reasonable when protein is restrained, without restraint, it would be safer to use a longer coordinate saving interval and correspondingly long simulation time, which were 5 ps and 400 ns (after the first 100 ns of the 500-ns simulation), respectively, in our analysis (Methods).

## 4 Conclusion

Present results quantitatively elucidate the heterogeneous and dynamic nature of surface hydration and its contribution to the desolvation energy of ligand binding. The residence time of surface water is typically in the picosecond time scale,^11^ though it varies over a wide range.^33,35,105^ During hundreds of nanoseconds of simulation time, there will be many exchange events of water molecules between the surface and the bulk, so that a lower free energy is associated with locations with a higher water density, i.e., where water is more stable (eq 1). It should be noted that this stability does not necessarily imply a longer residence time. The residence time of a surface-bound water molecule is a kinetic property determined by the height of the energy barrier that must be overcome to detach from the surface. On the other hand, the density-based solvation free energy (eq 1) is determined by the likelihood of visitation of a given cell by a water molecule. Since the small sizes of cubic cells we used can accommodate only one water oxygen, a higher water density in a cell does not mean more densely packed water molecules, but it instead indicates the cell is occupied by a water oxygen in a greater fraction of coordinate frames, where the associated free energy is lower. Thus, hydration sites that differ in water residence time can have similar solvation free energy.

A salient feature of the hydration shell that we measured is its fragmented coverage of the surface, which depends on the types of amino acids comprising the surface, surface topography, and, importantly, the mobility of the surface. Reduction in the extent of the first hydration shell and its water density upon ligand binding leads to desolvation penalty. In particular, the coupling between protein conformational dynamics and the hydration shell causes an allosteric effect where water around entropy hotspot residues^77^ that are located away from the ligand-binding interface contributes significantly to the desolvation penalty. Other remote sites may also involve increase or decrease in desolvation penalty, suggesting that nonlocal effects of ligand binding on surface hydration may be a general feature, for which conformational motion of the protein is crucial.

In our simulation, we used TIP3P water models associated with the CHARMM param36 force field.^81,82^ While there are water models with overall improved accuracy,^106^ compatibility with a given protein force field needs further examination. The surface water density map is determined mainly by the interaction with protein atoms and transient structural arrangements that are not heavily influenced by the differences among water models in current use. We thus expect our main conclusions regarding the heterogeneity and nonlocality of surface hydration not to depend on the water model in any major way.

Greater coverage of the surface water in simulations where protein atoms are restrained (Figure 9a,b) indicates that the heterogeneity in surface hydration is the result of local dynamics of amino acids. The nSH3 domain used in the present study is stably folded, so that a “blurring” effect, namely reduction in water density due to the motion of flexible regions relative to the orientational reference,^100^ is not significant, as seen by the linear correlation between Δ*G*_solv_ and Δ*G*_solv_^local^ (Figure 8). At the level of individual residues, protein surfaces are not smooth but have features on the length scale of several Ångströms, which is comparable to the size of a water molecule. Thus, the configuration of surface water molecules must change as the protein surface moves. In regions where nearby amino acid side chains differ in mobility, such as entropy hotspots, configurations or patterns of surface water molecules are less defined as side chains move relative to each other, resulting in a lower water density.

All of the entropy hotspot residues (Figure 7) except for Y190 that is near the C-terminus of nSH3 have low C_α_-based RMSF (less than 0.5 Å; Tables S2 and S3). Yet, they consistently have a large positive desolvation penalty regardless of whether local or global references are used for the water density map (Figure 8), which further highlights the nonlocal effect of side chain motion on surface hydration upon ligand binding. Side chain mobility of these hotspots increases because ligand binding leads to a cascade of changes in lateral contacts among surface residues.^77^ Given the wide range of involvement of the SH3 domain in signaling,^75^ such remote residues may offer additional ways to fine-tune ligand binding.

Comparing Δ*G*_solv_ and Δ*G*_solv_^local^ for individual residues (Figure 8) can be informative for identifying residues whose behaviors change substantially when a ligand binds. A similar approach may be applied to multidomain proteins undergoing hinge motion. By constructing water density maps with individual domains as a respective reference for coordinate alignment and comparing residue-level solvation energies, e.g., between bent vs extended conformations, it may be possible to identify residues that undergo large changes in hydration at the hinge region, which would be a subject of future studies.

We demonstrated the advantage of our density-based method over GIST, in that our method does not require protein atoms to be restrained, yields solvation energies in a more reasonable range (Figure 10b), and is less demanding in terms of sampling. After all, protein and water form a strongly coupled and dynamic system, so that considering thermodynamic properties of water separately would only be an approximation, where various computational methods capture related as well as different aspects. To our knowledge, this is the first approach to account for the dynamic interplay between protein motion and surface hydration in ligand binding. As such, we expect that it will be broadly applicable to studying molecular recognition and ligand design.

## Supporting Information Available

Table S1, dependence of calculated desolvation free energy on resolution of hydration map; Table S2, residue-level changes in desolvation energy for nSH3:PRM^cAbl^; Table S3, residue-level changes in desolvation energy for nSH3:PRM^NS1^; Table S4, residue-level changes in desolvation energy for nSH3:PRM^cAbl^ in restrained simulations; Table S5, residue-level changes in desolvation energy for nSH3:PRM^NS1^ in restrained simulations (PDF)

## References

[ref1] Papoian G. A.; Ulander J.; Eastwood M. P.; Luthey-Schulten Z.; Wolynes P. G. Water in protein structure prediction. Proc. Natl. Acad. Sci. U.S.A. 2004, 101, 3352–3357. 10.1073/pnas.0307851100. 14988499 PMC373465

[ref2] Ball P. Water as an active constituent in cell biology. Chem. Rev. 2008, 108, 74–108. 10.1021/cr068037a. 18095715

[ref3] Duboué-Dijon E.; Fogarty A. C.; Hynes J. T.; Laage D. Dynamical disorder in the DNA hydration shell. J. Am. Chem. Soc. 2016, 138, 7610–7620. 10.1021/jacs.6b02715. 27240107

[ref4] Bellissent-Funel M.-C.; Hassanali A.; Havenith M.; Henchman R.; Pohl P.; Sterpone F.; van der Spoel D.; Xu Y.; Garcia A. E. Water determines the structure and dynamics of proteins. Chem. Rev. 2016, 116, 7673–7697. 10.1021/acs.chemrev.5b00664. 27186992 PMC7116073

[ref5] Maurer M.; Oostenbrink C. Water in protein hydration and ligand recognition. J. Mol. Recognit. 2019, 32, e2810 10.1002/jmr.2810. 31456282 PMC6899928

[ref6] Burling F. T.; Weis W. I.; Flaherty K. M.; Brünger A. T. Direct observation of protein solvation and discrete disorder with experimental crystallographic phases. Science 1996, 271, 72–77. 10.1126/science.271.5245.72. 8539602

[ref7] Svergun D.; Richard S.; Koch M.; Sayers Z.; Kuprin S.; Zaccai G. Protein hydration in solution: experimental observation by x-ray and neutron scattering. Proc. Natl. Acad. Sci. U.S.A. 1998, 95, 2267–2272. 10.1073/pnas.95.5.2267. 9482874 PMC19315

[ref8] Merzel F.; Smith J. C. Is the first hydration shell of lysozyme of higher density than bulk water?. Proc. Natl. Acad. Sci. U.S.A. 2002, 99, 5378–5383. 10.1073/pnas.082335099. 11959992 PMC122777

[ref9] Ravikumar K. M.; Hwang W. Role of Hydration Force in the Self-Assembly of Collagens and Amyloid Steric Zipper Filaments. J. Am. Chem. Soc. 2011, 133, 11766–11773. 10.1021/ja204377y. 21692533 PMC3145038

[ref10] Marchi M.; Sterpone F.; Ceccarelli M. Water rotational relaxation and diffusion in hydrated lysozyme. J. Am. Chem. Soc. 2002, 124, 6787–6791. 10.1021/ja025905m. 12047201

[ref11] Laage D.; Elsaesser T.; Hynes J. T. Water dynamics in the hydration shells of biomolecules. Chem. Rev. 2017, 117, 10694–10725. 10.1021/acs.chemrev.6b00765. 28248491 PMC5571470

[ref12] Lu Y.; Wang R.; Yang C.-Y.; Wang S. Analysis of ligand-bound water molecules in high-resolution crystal structures of protein- ligand complexes. J. Chem. Inf. Model. 2007, 47, 668–675. 10.1021/ci6003527. 17266298

[ref13] Michel J.; Tirado-Rives J.; Jorgensen W. L. Energetics of displacing water molecules from protein binding sites: consequences for ligand optimization. J. Am. Chem. Soc. 2009, 131, 15403–15411. 10.1021/ja906058w. 19778066 PMC2783447

[ref14] de Beer S.; Vermeulen N. P.; Oostenbrink C. The role of water molecules in computational drug design. Curr. Top. Med. Chem. 2010, 10, 55–66. 10.2174/156802610790232288. 19929830

[ref15] Bortolato A.; Tehan B. G.; Bodnarchuk M. S.; Essex J. W.; Mason J. S. Water network perturbation in ligand binding: Adenosine A_2A_ antagonists as a case study. J. Chem. Info. Model. 2013, 53, 1700–1713. 10.1021/ci4001458. 23725291

[ref16] Wahl J.; Smieško M. Thermodynamic insight into the effects of water displacement and rearrangement upon ligand modifications using molecular dynamics simulations. ChemMedChem. 2018, 13, 1325–1335. 10.1002/cmdc.201800093. 29726604

[ref17] Darby J. F.; Hopkins A. P.; Shimizu S.; Roberts S. M.; Brannigan J. A.; Turkenburg J. P.; Thomas G. H.; Hubbard R. E.; Fischer M. Water networks can determine the affinity of ligand binding to proteins. J. Am. Chem. Soc. 2019, 141, 15818–15826. 10.1021/jacs.9b06275. 31518131

[ref18] Spitaleri A.; Zia S. R.; Di Micco P.; Al-Lazikani B.; Soler M. A.; Rocchia W. Tuning Local Hydration Enables a Deeper Understanding of Protein–Ligand Binding: The PP1-Src Kinase Case. J. Phys. Chem. Lett. 2021, 12, 49–58. 10.1021/acs.jpclett.0c03075. 33300337 PMC7812613

[ref19] Nakasako M. Water–protein interactions from high–resolution protein crystallography. Philos. Trans. R. Soc. London B Biol. Sci. 2004, 359, 1191–1206. 10.1098/rstb.2004.1498. 15306376 PMC1693410

[ref20] Tarek M.; Tobias D. J. The dynamics of protein hydration water: a quantitative comparison of molecular dynamics simulations and neutron-scattering experiments. Biophys. J. 2000, 79, 3244–3257. 10.1016/S0006-3495(00)76557-X. 11106628 PMC1301199

[ref21] Stanley C.; Krueger S.; Parsegian V. A.; Rau D. C. Protein structure and hydration probed by SANS and osmotic stress. Biophys. J. 2008, 94, 2777–2789. 10.1529/biophysj.107.122697. 18178651 PMC2267150

[ref22] Combet S.; Zanotti J.-M. Further evidence that interfacial water is the main “driving force” of protein dynamics: a neutron scattering study on perdeuterated C-phycocyanin. Phys. Chem. Chem. Phys. 2012, 14, 4927–4934. 10.1039/c2cp23725c. 22388956

[ref23] Oleinikova A.; Sasisanker P.; Weingärtner H. What can really be learned from dielectric spectroscopy of protein solutions? A case study of ribonuclease A. J. Phys. Chem. B 2004, 108, 8467–8474. 10.1021/jp049618b.

[ref24] Yagihara S.; Saito H.; Sugimoto H.; Kawaguchi T.; Fukuzaki M.; Igarashi T.; Hoshi M.; Nakamura K. Evaluation of water structures in cotton cloth by fractal analysis with broadband dielectric spectroscopy. J. Mater. Sci. 2021, 56, 17844–17859. 10.1007/s10853-021-06419-7.

[ref25] King J. T.; Kubarych K. J. Site-specific coupling of hydration water and protein flexibility studied in solution with ultrafast 2D-IR spectroscopy. J. Am. Chem. Soc. 2012, 134, 18705–18712. 10.1021/ja307401r. 23101613

[ref26] King J. T.; Arthur E. J.; Brooks C. L. III; Kubarych K. J. Crowding induced collective hydration of biological macromolecules over extended distances. J. Am. Chem. Soc. 2014, 136, 188–194. 10.1021/ja407858c. 24341684 PMC3983708

[ref27] Salehi S. M.; Meuwly M. Site-selective dynamics of azidolysozyme. J. Chem. Phys. 2021, 154, 165101 10.1063/5.0047330. 33940854

[ref28] Otting G.; Liepinsh E.; Wuthrich K. Protein hydration in aqueous solution. Science 1991, 254, 974–980. 10.1126/science.1948083. 1948083

[ref29] Halle B. Protein hydration dynamics in solution: a critical survey. Philos. Trans. R. Soc. London B Biol. Sci. 2004, 359, 1207–1224. 10.1098/rstb.2004.1499. 15306377 PMC1693401

[ref30] Nucci N. V.; Pometun M. S.; Wand A. J. Mapping the hydration dynamics of ubiquitin. J. Am. Chem. Soc. 2011, 133, 12326–12329. 10.1021/ja202033k. 21761828 PMC3155818

[ref31] Armstrong B. D.; Choi J.; López C.; Wesener D. A.; Hubbell W.; Cavagnero S.; Han S. Site-specific hydration dynamics in the nonpolar core of a molten globule by dynamic nuclear polarization of water. J. Am. Chem. Soc. 2011, 133, 5987–5995. 10.1021/ja111515s. 21443207 PMC3095581

[ref32] Kaieda S.; Halle B. Internal water and microsecond dynamics in myoglobin. J. Phys. Chem. B 2013, 117, 14676–14687. 10.1021/jp409234g. 24195787 PMC3966298

[ref33] Fenwick R. B.; Oyen D.; Dyson H. J.; Wright P. E. Slow dynamics of tryptophan–water networks in proteins. J. Am. Chem. Soc. 2018, 140, 675–682. 10.1021/jacs.7b09974. 29256600 PMC5771866

[ref34] Jorge C.; Marques B. S.; Valentine K. G.; Wand A. J. Methods Enzymol.; Elsevier: 2019; Vol. 615, pp 77–101, 10.1016/bs.mie.2018.09.040. 30638541 PMC6358200

[ref35] Houston P.; Macro N.; Kang M.; Chen L.; Yang J.; Wang L.; Wu Z.; Zhong D. Ultrafast Dynamics of Water–Protein Coupled Motions around the Surface of Eye Crystallin. J. Am. Chem. Soc. 2020, 142, 3997–4007. 10.1021/jacs.9b13506. 31991083 PMC7261499

[ref36] Persson F.; Halle B. Transient access to the protein interior: Simulation versus NMR. J. Am. Chem. Soc. 2013, 135, 8735–8748. 10.1021/ja403405d. 23675835

[ref37] Amann-Winkel K.; Bellissent-Funel M.-C.; Bove L. E.; Loerting T.; Nilsson A.; Paciaroni A.; Schlesinger D.; Skinner L. X-ray and neutron scattering of water. Chem. Rev. 2016, 116, 7570–7589. 10.1021/acs.chemrev.5b00663. 27195477

[ref38] Frauenfelder H.; Fenimore P. W.; Chen G.; McMahon B. H. Protein folding is slaved to solvent motions. Proc. Natl. Acad. Sci. U.S.A. 2006, 103, 15469–15472. 10.1073/pnas.0607168103. 17030792 PMC1592535

[ref39] Mukherjee S.; Mondal S.; Bagchi B. Mechanism of solvent control of protein dynamics. Phys. Rev. Lett. 2019, 122, 058101 10.1103/PhysRevLett.122.058101. 30822020

[ref40] Fogarty A. C.; Laage D. Water dynamics in protein hydration shells: the molecular origins of the dynamical perturbation. J. Phys. Chem. B 2014, 118, 7715–7729. 10.1021/jp409805p. 24479585 PMC4103960

[ref41] Schirò G.; Weik M. Role of hydration water in the onset of protein structural dynamics. J. Phys.: Condens. Matter 2019, 31, 463002 10.1088/1361-648X/ab388a. 31382251

[ref42] Zwanzig R. W. High-temperature equation of state by a perturbation method. I. Nonpolar gases. J. Chem. Phys. 1954, 22, 1420–1426. 10.1063/1.1740409.

[ref43] Kirkwood J. G. Statistical mechanics of fluid mixtures. J. Chem. Phys. 1935, 3, 300–313. 10.1063/1.1749657.

[ref44] Ge Y.; Melling O. J.; Dong W.; Essex J. W.; Mobley D. L. Enhancing sampling of water rehydration upon ligand binding using variants of grand canonical Monte Carlo. J. Comput. Aid. Mol. Des. 2022, 36, 767–779. 10.1007/s10822-022-00479-w. PMC9869699 36198874

[ref45] Peter C.; Oostenbrink C.; Van Dorp A.; Van Gunsteren W. F. Estimating entropies from molecular dynamics simulations. J. Chem. Phys. 2004, 120, 2652–2661. 10.1063/1.1636153. 15268408

[ref46] Huggins D. J. Quantifying the entropy of binding for water molecules in protein cavities by computing correlations. Biophys. J. 2015, 108, 928–936. 10.1016/j.bpj.2014.12.035. 25692597 PMC4336375

[ref47] Khanna V.; Monroe J. I.; Doherty M. F.; Peters B. Performing solvation free energy calculations in LAMMPS using the decoupling approach. J. Comput. Aided Mol. Des. 2020, 34, 641–646. 10.1007/s10822-020-00303-3. 32112288

[ref48] Kollman P. A.; Massova I.; Reyes C.; Kuhn B.; Huo S.; Chong L.; Lee M.; Lee T.; Duan Y.; Wang W.; Donini O.; Cieplak P.; Srinivasan J.; Case D. A.; Cheatham T. E. Calculating structures and free energies of complex molecules: combining molecular mechanics and continuum models. Acc. Chem. Res. 2000, 33, 889–897. 10.1021/ar000033j. 11123888

[ref49] Lee M. S.; Salsbury F. R. Jr; Brooks C. L. III Novel generalized Born methods. J. Chem. Phys. 2002, 116, 10606–10614. 10.1063/1.1480013.

[ref50] Zoete V.; Meuwly M.; Karplus M. Study of the insulin dimerization: binding free energy calculations and per-residue free energy decomposition. Proteins 2005, 61, 79–93. 10.1002/prot.20528. 16080143

[ref51] Park J.; Kahng B.; Hwang W. Thermodynamic Selection of Steric Zipper Patterns in the Amyloid Cross-β Spine. PLoS Comput. Biol. 2009, 5, e1000492 10.1371/journal.pcbi.1000492. 19730673 PMC2723932

[ref52] Roux B. Continuum electrostatic behavior of a 3D-RISM theory. J. Phys. Chem. B 2020, 124, 7444–7451. 10.1021/acs.jpcb.0c05519. 32697579 PMC7568820

[ref53] Kleinjung J.; Fraternali F. Design and application of implicit solvent models in biomolecular simulations. Curr. Opin. Struct. Biol. 2014, 25, 126–134. 10.1016/j.sbi.2014.04.003. 24841242 PMC4045398

[ref54] Yang P.-K. Modifying Poisson equation for near-solute dielectric polarization and solvation free energy. Chem. Phys. 2016, 472, 229–240. 10.1016/j.chemphys.2016.02.016.

[ref55] Harris R. C.; Pettitt B. M. Effects of geometry and chemistry on hydrophobic solvation. Proc. Natl. Acad. Sci. U.S.A. 2014, 111, 14681–14686. 10.1073/pnas.1406080111. 25258413 PMC4205605

[ref56] Harris R. C.; Pettitt B. M. Examining the assumptions underlying continuum-solvent models. J. Chem. Theory Comp. 2015, 11, 4593–4600. 10.1021/acs.jctc.5b00684. PMC5340276 26574250

[ref57] Lazaridis T.; Paulaitis M. E. Entropy of hydrophobic hydration: a new statistical mechanical formulation. J. Phys. Chem. 1992, 96, 3847–3855. 10.1021/j100188a051.

[ref58] Lazaridis T. Inhomogeneous fluid approach to solvation thermodynamics. 1. Theory. J. Phys. Chem. B 1998, 102, 3531–3541. 10.1021/jp9723574.

[ref59] Lazaridis T. Inhomogeneous fluid approach to solvation thermodynamics. 2. Applications to simple fluids. J. Phys. Chem. B 1998, 102, 3542–3550. 10.1021/jp972358w.

[ref60] Li Z.; Lazaridis T. Thermodynamic contributions of the ordered water molecule in HIV-1 protease. J. Am. Chem. Soc. 2003, 125, 6636–6637. 10.1021/ja0299203. 12769565

[ref61] Huggins D. J.; Marsh M.; Payne M. C. Thermodynamic properties of water molecules at a protein–protein interaction surface. J. Chem. Theory Comp. 2011, 7, 3514–3522. 10.1021/ct200465z. PMC3924879 24554921

[ref62] Nguyen C. N.; Kurtzman Young T.; Gilson M. K. Grid inhomogeneous solvation theory: hydration structure and thermodynamics of the miniature receptor cucurbit [7] uril. J. Chem. Phys. 2012, 137, 044101 10.1063/1.4733951. 22852591 PMC3416872

[ref63] Kraml J.; Kamenik A. S.; Waibl F.; Schauperl M.; Liedl K. R. Solvation free energy as a measure of hydrophobicity: application to serine protease binding interfaces. J. Chem. Theory Comp. 2019, 15, 5872–5882. 10.1021/acs.jctc.9b00742. PMC7032847 31589427

[ref64] Irwin B. W.; Vukovic S.; Payne M. C.; Huggins D. J. Large-scale study of hydration environments through hydration sites. J. Phys. Chem. B 2019, 123, 4220–4229. 10.1021/acs.jpcb.9b02490. 31025866

[ref65] Heinz L. P.; Grubmüller H. Computing spatially resolved rotational hydration entropies from atomistic simulations. J. Chem. Theory Comp. 2020, 16, 108–118. 10.1021/acs.jctc.9b00926. 31822062

[ref66] Dunitz J. D. The entropic cost of bound water in crystals and biomolecules. Science 1994, 264, 670–671. 10.1126/science.264.5159.670. 17737951

[ref67] White R. P.; Meirovitch H. A simulation method for calculating the absolute entropy and free energy of fluids: Application to liquid argon and water. Proc. Natl. Acad. Sci. U.S.A. 2004, 101, 9235–9240. 10.1073/pnas.0308197101. 15197270 PMC438959

[ref68] General I. J.; Dragomirova R.; Meirovitch H. New method for calculating the absolute free energy of binding: The effect of a mobile loop on the avidin/biotin complex. J. Phys. Chem. B 2011, 115, 168–175. 10.1021/jp1076752. 21158467 PMC3042141

[ref69] Yousefi R.; Lynch G. C.; Galbraith M.; Pettitt B. M. Contributions of higher-order proximal distribution functions to solvent structure around proteins. J. Chem. Phys. 2021, 155, 104110 10.1063/5.0062580. 34525817 PMC8439718

[ref70] Teng X.; Hwang W. Effect of methylation on local mechanics and hydration structure of DNA. Biophys. J. 2018, 114, 1791–1803. 10.1016/j.bpj.2018.03.022. 29694859 PMC5937226

[ref71] Feller S. M. Crk family adaptors-signalling complex formation and biological roles. Oncogene 2001, 20, 6348–6371. 10.1038/sj.onc.1204779. 11607838

[ref72] Bhatt V. S.; Zeng D.; Krieger I.; Sacchettini J. C.; Cho J.-H. Binding Mechanism of the N-Terminal SH3 Domain of CrkII and Proline-Rich Motifs in cAbl. Biophys. J. 2016, 110, 2630–2641. 10.1016/j.bpj.2016.05.008. 27332121 PMC4919510

[ref73] Shen Q.; Zeng D.; Zhao B.; Bhatt V. S.; Li P.; Cho J.-H. The Molecular Mechanisms Underlying the Hijack of Host Proteins by the 1918 Spanish Influenza Virus. ACS Chem. Biol. 2017, 12, 1199–1203. 10.1021/acschembio.7b00168. 28368102

[ref74] Shen Q.; Shi J.; Zeng D.; Zhao B.; Li P.; Hwang W.; Cho J.-H. Molecular Mechanisms of Tight Binding through Fuzzy Interactions. Biophys. J. 2018, 114, 1313–1320. 10.1016/j.bpj.2018.01.031. 29590589 PMC5883614

[ref75] Teyra J.; Huang H.; Jain S.; Guan X.; Dong A.; Liu Y.; Tempel W.; Min J.; Tong Y.; Kim P. M.; Bader G. D.; Sidhu S. S. Comprehensive analysis of the human SH3 domain family reveals a wide variety of non-canonical specificities. Structure 2017, 25, 1598–1610. 10.1016/j.str.2017.07.017. 28890361

[ref76] Opitz R.; Müller M.; Reuter C.; Barone M.; Soicke A.; Roske Y.; Piotukh K.; Huy P.; Beerbaum M.; Wiesner B.; Beyermann M.; Schmieder P.; Freund C.; Volkmer R.; Oschkinat H.; Schmalz H.-G.; Kühhne R. A modular toolkit to inhibit proline-rich motif–mediated protein–protein interactions. Proc. Natl. Acad. Sci. U.S.A. 2015, 112, 5011–5016. 10.1073/pnas.1422054112. 25848013 PMC4413326

[ref77] Shi J.; Shen Q.; Cho J.-H.; Hwang W. Entropy Hotspots for the Binding of Intrinsically Disordered Ligands to a Receptor Domain. Biophys. J. 2020, 118, 2502–2512. 10.1016/j.bpj.2020.03.026. 32311315 PMC7231926

[ref78] Balius T. E.; Fischer M.; Stein R. M.; Adler T. B.; Nguyen C. N.; Cruz A.; Gilson M. K.; Kurtzman T.; Shoichet B. K. Testing inhomogeneous solvation theory in structure-based ligand discovery. Proc. Natl. Acad. Sci. U.S.A. 2017, 114, E6839–E6846. 10.1073/pnas.1703287114. 28760952 PMC5565424

[ref79] Brooks B. R.; Bruccoleri R. E.; Olafson B. D.; States D. J.; Swaminathan S.; Karplus M. CHARMM: A program for macromolecular energy, minimization, and dynamics calculations. J. Comput. Chem. 1983, 4, 187–217. 10.1002/jcc.540040211.

[ref80] Brooks B. R.; Brooks C. L. III; Mackerell A. D. Jr.; Nilsson L.; Petrella R. J.; Roux B.; Won Y.; Archontis G.; Bartels C.; Boresch S.; Caflisch A.; Caves L.; Cui Q.; Dinner A. R.; Feig M.; Fischer S.; Gao J.; Hodoscek M.; Im W.; Kuczera K.; Lazaridis T.; Ma J.; Ovchinnikov V.; Paci E.; Pastor R. W.; Post C. B.; Pu J. Z.; Schaefer M.; Tidor B.; Venable R. M.; Woodcock H. L.; Wu X.; Yang W.; York D. M.; Karplus M. CHARMM: the biomolecular simulation program. J. Comput. Chem. 2009, 30, 1545–1614. 10.1002/jcc.21287. 19444816 PMC2810661

[ref81] Huang J.; MacKerell A. D. Jr CHARMM36 all-atom additive protein force field: Validation based on comparison to NMR data. J. Comput. Chem. 2013, 34, 2135–2145. 10.1002/jcc.23354. 23832629 PMC3800559

[ref82] Jorgensen W.; Chandrasekhar J.; Madura J. D. Comparison of simple potential functions for simulating liquid water. J. Chem. Phys. 1983, 79, 926–935. 10.1063/1.445869.

[ref83] Mark P.; Nilsson L. Structure and dynamics of the TIP3P, SPC, and SPC/E water models at 298 K. J. Phys. Chem. A 2001, 105, 9954–9960. 10.1021/jp003020w.

[ref84] Pettersen E. F.; Goddard T. D.; Huang C. C.; Couch G. S.; Greenblatt D. M.; Meng E. C.; Ferrin T. E. UCSF Chimera-A visualization system for exploratory research and analysis. J. Comput. Chem. 2004, 25, 1605–1612. 10.1002/jcc.20084. 15264254

[ref85] Humphrey W.; Dalke A.; Schulten K. VMD: Visual Molecular Dynamics. J. Mol. Graphics. 1996, 14, 33–38. 10.1016/0263-7855(96)00018-5. 8744570

[ref86] Bagchi B. Water dynamics in the hydration layer around proteins and micelles. Chem. Rev. 2005, 105, 3197–3219. 10.1021/cr020661+. 16159150

[ref87] Im W.; Lee M. S.; Brooks C. L. Generalized born model with a simple smoothing function. J. Comput. Chem. 2003, 24, 1691–1702. 10.1002/jcc.10321. 12964188

[ref88] King B.; Tidor B. MIST: Maximum Information Spanning Trees for dimension reduction of biological data sets. Bioinformatics 2009, 25, 1165–1172. 10.1093/bioinformatics/btp109. 19261718 PMC2672626

[ref89] King B.; Silver N.; Tidor B. Efficient Calculation of Molecular Configurational Entropies Using an Information Theoretic Approximation. J. Phys. Chem. B 2012, 116, 2891–2904. 10.1021/jp2068123. 22229789 PMC3465721

[ref90] Bates D. M.; Watts D. G. Nonlinear Regression Analysis and Its Applications; Wiley: 1988.

[ref91] Genheden S.; Ryde U. The MM/PBSA and MM/GBSA methods to estimate ligand-binding affinities. Exp. Op. Drug Disc. 2015, 10, 449–461. 10.1517/17460441.2015.1032936. PMC4487606 25835573

[ref92] Wang C.; Pawley N. H.; Nicholson L. K. The role of backbone motions in ligand binding to the c-Src SH3 domain. J. Mol. Biol. 2001, 313, 873–887. 10.1006/jmbi.2001.5083. 11697910

[ref93] Hou T.; Chen K.; McLaughlin W. A.; Lu B.; Wang W. Computational analysis and prediction of the binding motif and protein interacting partners of the Abl SH3 domain. PLoS. Comput. Biol. 2006, 2, e1 10.1371/journal.pcbi.0020001. 16446784 PMC1356089

[ref94] Woo H.-J.; Roux B. Calculation of absolute protein–ligand binding free energy from computer simulations. Proc. Natl. Acad. Sci. U.S.A. 2005, 102, 6825–6830. 10.1073/pnas.0409005102. 15867154 PMC1100764

[ref95] Doig A.; Sternberg M. J. E. Side-chain conformational entropy in protein folding. Protein Sci. 1995, 4, 2247–2251. 10.1002/pro.5560041101. 8563620 PMC2143028

[ref96] Kasinath V.; Sharp K. A.; Wand A. J. Microscopic Insights into the NMR Relaxation-Based Protein Conformational Entropy Meter. J. Am. Chem. Soc. 2013, 135, 15092–15100. 10.1021/ja405200u. 24007504 PMC3821934

[ref97] Roux B.; Simonson T. Implicit solvent models. Biophys. Chem. 1999, 78, 1–20. 10.1016/S0301-4622(98)00226-9. 17030302

[ref98] Rath A.; Davidson A. R.; Deber C. M. The structure of ”unstructured” regions in peptides and proteins: Role of the polyproline II helix in protein folding and recognition. Biopolymers 2005, 80, 179–185. 10.1002/bip.20227. 15700296

[ref99] Lee B.; Richards F. M. The interpretation of protein structures: estimation of static accessibility. J. Mol. Biol. 1971, 55, 379–IN4. 10.1016/0022-2836(71)90324-X. 5551392

[ref100] Henchman R. H.; McCammon J. A. Extracting hydration sites around proteins from explicit water simulations. J. Comput. Chem. 2002, 23, 861–869. 10.1002/jcc.10074. 11984847

[ref101] Ramsey S.; Nguyen C.; Salomon-Ferrer R.; Walker R. C.; Gilson M. K.; Kurtzman T. Solvation thermodynamic mapping of molecular surfaces in AmberTools: GIST. J Comput Chem. 2016, 37, 2029 10.1002/jcc.24417. 27317094 PMC5052087

[ref102] Abraham F. The interfacial density profile of a Lennard-Jones fluid in contact with a (100) Lennard-Jones wall and its relationship to idealized fluid/wall systems: A Monte Carlo simulation. J. Chem. Phys. 1978, 68, 3713–3716. 10.1063/1.436229.

[ref103] Merzel F.; Smith J. C. Is the first hydration shell of lysozyme of higher density than bulk water?. Proc. Natl. Acad. Sci. U.S.A. 2002, 99, 5378–5383. 10.1073/pnas.082335099. 11959992 PMC122777

[ref104] Roe D. R.; Cheatham T. E. III PTRAJ and CPPTRAJ: software for processing and analysis of molecular dynamics trajectory data. J. Chem. Theory Comp. 2013, 9, 3084–3095. 10.1021/ct400341p. 26583988

[ref105] Mondal S.; Mukherjee S.; Bagchi B. Origin of diverse time scales in the protein hydration layer solvation dynamics: A simulation study. J. Chem. Phys. 2017, 147, 154901 10.1063/1.4995420. 29055291

[ref106] Xiong Y.; Onufriev A. V. Exploring optimization strategies for improving explicit water models: Rigid n-point model and polarizable model based on Drude oscillator. PLoS One 2019, 14, e0224991 10.1371/journal.pone.0224991. 31725740 PMC6855648

