# Supplementary material for: Heterogeneous and Allosteric Role of Surface Hydration for Protein–Ligand Binding
Source: J Chem Theory Comput. 2023 Feb 23;19(6):1875–87. doi: 10.1021/acs.jctc.2c00776 (PMC10848206; doi:10.1021/acs.jctc.2c00776)
Supplement: Supplementary file 1 — ct2c00776_si_001.pdf [file ct2c00776_si_001.pdf]

Supporting Information: Heterogeneous and Allosteric Role of Surface Hydration for Protein-Ligand Binding

Jie Shi,[†,‖] Jae-Hyun Cho,[‡] and Wonmuk Hwang[*,†,¶,§]

†*Department of Biomedical Engineering, Texas A&M University, College Station, Texas, 77843, USA*
‡*Department of Biochemistry and Biophysics, Texas A&M University, College Station, Texas, 77843, USA*
¶*Department of Materials Science and Engineering, Texas A&M University, College Station, Texas, 77843, USA*
§*Department of Physics and Astronomy, Texas A&M University, College Station, Texas, 77843, USA*
‖*Current addresses: Functional Genomics and Cellular Engineering, IBM Almaden Research Center, San Jose, CA 95120; USA, Center for Cellular Construction, San Francisco, CA 94158, USA*

E-mail: hwm@tamu.edu

Table S1: Dependence of the calculated desolvation free energy on the resolution of the hydration map. The resolution (in units of Å) was varied up to 1.4 Å, where up to 0.9 Å is shown. Energy terms are in kcal/mol. $N_{\rm cell}$: total number of cells used for calculation. $G_{\rm solv}$: From Eq. 1. $\Delta G_{\rm solv}$: the difference between the complex and the unliganded systems. $G^{\infty}_{\rm solv}$: asymptotic value for $G_{\rm solv}$ via exponential fits in Fig. 5. Uncertainties (in parentheses) at each resolution are measured by subsampling the trajectory every eighth frames (a total of eight sets). Uncertainty in $G^{\infty}_{\rm solv}$ is by nonlinear regression as explained in Fig. 5.

	Resolution	N_{cell}	$G_{\rm solv}$	$\Delta G_{\rm solv}$	$G^{\infty}_{\rm solv}$
	0.5	32845 (76)	-6477.9 (6.7)	—	
	0.6	19102 (38)	-3645.0 (4.7)	—	
Unbound nSH3	0.7	12129 (45)	-2246.0 (3.2)	—	-277.1 (46.3)
	0.8	8048 (35)	-1460.0 (3.6)	—	
	0.9	5708 (23)	-1006.1 (1.5)	—	
	0.5	4527 (47)	-419.3 (3.8)	—	
	0.6	2273 (29)	-212.9 (1.6)	—	
Unbound PRM^{cAbl}	0.7	1278 (24)	-122.9 (2.0)	—	-14.6 (3.2)
	0.8	788 (19)	-76.7 (1.2)	—	
	0.9	509 (22)	-49.4 (0.9)	—	
	0.5	7142 (48)	-903.4 (6.6)	—	
	0.6	3778 (46)	-487.4 (2.5)	—	
Unbound PRM^{NS1}	0.7	2234 (37)	-293.1 (2.0)	—	-36.5 (6.7)
	0.8	1385 (33)	-186.4 (1.3)	—	
	0.9	940 (15)	-126.3 (1.0)	—	
	0.5	32524 (58)	-6293.6 (10.3)	603.6 (20.8)	
	0.6	18738 (81)	-3531.4 (6.1)	326.5 (12.4)	
cAbl Complex	0.7	11807 (42)	-2167.4 (3.4)	201.6 (8.5)	-264.4 (46.1)
	0.8	7883 (31)	-1414.3 (2.2)	122.4 (7.0)	
	0.9	5539 (20)	- 974.7 (1.7)	80.8 (4.0)	
	0.5	34234 (91)	-6881.7 (10.6)	499.6 (24.0)	
	0.6	19741 (49)	-3877.4 (6.1)	255.0 (13.3)	
NS1 Complex	0.7	12423 (42)	-2379.8 (5.3)	159.3 (10.4)	-293.2 (48.9)
	0.8	8279 (24)	-1552.7 (2.0)	93.7 (6.9)	
	0.9	5862 (12)	-1071.0 (1.8)	61.3 (4.3)	

Table S2: Residue-level changes in the desolvation energy for nSH3:PRM^{cAbl}. Data are shown in the descending order of ΔG_{solv} (desolvation penalty; in kcal/mol). Residues in the ligand-binding pocket are listed in the lower section, separated by double horizontal lines. $N^u_{\mathrm{cell}}/N^b_{\mathrm{cell}}$: Number of cells included in Eq. 1 without/with the bound ligand. Uncertainties (in parentheses) are measured by subsampling the trajectory, as explained in Table S1. $\Delta G^{\mathrm{local}}_{\mathrm{solv}}$: The same calculation except that coordinate frames are aligned locally with the backbone heavy atoms and C_β atom for each residue as reference. C_α RMSF (in Å): Root-mean-square fluctuation of each C_α atom of nSH3 in the ligand-bound complex.

Residue #	N^u_{cell}	N^b_{cell}	ΔG_{solv}	$\Delta G^{\mathrm{local}}_{\mathrm{solv}}$	C_α RMSF
162	590(6.44)	574(7.34)	19.35(1.71)	25.23	0.42
155	568(8.12)	479(6.29)	13.42(1.19)	30.97	0.46
135	407(7.13)	420(9.43)	11.29(1.59)	7.56	0.62
164	573(4.62)	531(6.14)	10.93(1.68)	3.28	0.51
138	425(7.82)	437(9.33)	10.55(1.45)	17.56	0.35
136	444(8.00)	421(5.54)	9.87(1.44)	6.46	0.43
160	399(7.09)	383(4.17)	9.02(1.80)	4.81	0.36
161	118(3.70)	102(1.73)	8.04(0.81)	3.73	0.36
190	403(8.05)	462(6.73)	7.35(1.49)	-1.71	0.67
148	717(12.70)	673(7.94)	5.95(1.94)	0.46	0.97
179	623(8.57)	604(8.75)	5.90(1.79)	6.12	0.48
134	228(4.36)	270(7.85)	5.28(1.29)	0.29	3.18
173	240(3.23)	228(3.85)	5.15(1.30)	1.22	0.36
152	386(3.34)	350(3.28)	4.80(1.35)	2.36	0.38
163	447(5.04)	417(3.80)	3.86(0.92)	1.71	0.47
184	158(2.00)	154(2.30)	3.25(0.48)	6.12	0.39
191	499(7.63)	572(8.71)	3.20(1.70)	-5.12	1.78
182	10(0.46)	7(0.46)	3.06(0.15)	3.88	0.32
180	94(1.51)	96(2.56)	2.44(0.61)	0.79	0.35
177	347(3.93)	325(7.81)	1.20(1.82)	1.18	0.75
171	223(2.25)	214(4.56)	0.66(0.79)	-2.12	0.34
137	20(0.92)	22(1.69)	0.36(0.25)	0.93	0.36
159	68(1.58)	67(1.96)	0.26(0.56)	0.52	0.35
153	111(2.92)	106(1.69)	0.14(0.58)	3.86	0.34
172	3(0.83)	3(0.00)	−0.33(0.11)	-0.33	0.35
156	301(6.23)	309(4.21)	−0.57(0.89)	2.67	0.46
189	242(5.46)	330(10.14)	−1.15(1.60)	12.53	0.60
151	68(1.85)	67(3.44)	−1.26(0.61)	-0.25	0.32
139	40(1.77)	50(0.89)	−1.73(0.26)	-0.30	0.33
158	338(4.60)	364(4.10)	−2.52(1.32)	4.51	0.38
187	84(2.62)	89(1.85)	−2.72(0.57)	-1.85	0.38
178	532(4.53)	495(8.00)	−2.78(1.62)	-12.45	0.57
174	218(1.04)	214(4.98)	−3.27(0.90)	-2.21	0.47
145	196(4.28)	179(1.85)	−3.29(0.74)	-3.38	0.61
175	548(5.63)	540(4.00)	−3.51(2.25)	-0.80	0.75
157	253(3.11)	269(2.73)	−5.75(0.91)	1.40	0.43
188	264(3.69)	317(5.06)	−5.94(1.50)	-1.41	0.47
170	228(4.60)	263(6.21)	−7.74(0.88)	-9.71	0.34
176	754(6.92)	739(7.14)	−8.24(2.06)	-5.53	0.83
140	342(4.88)	356(4.10)	−8.27(1.12)	-12.19	0.42
165	495(6.21)	484(5.64)	−9.32(1.13)	-12.09	0.62
154	532(7.57)	590(10.83)	−14.25(1.83)	-16.21	0.43
169	497(7.40)	145(3.63)	51.63(0.85)	59.81	0.38
186	370(6.12)	83(2.39)	43.00(0.54)	44.03	0.37
146	647(8.49)	481(5.36)	40.09(1.93)	19.79	0.91
185	409(5.94)	255(4.42)	34.07(1.28)	44.60	0.45
183	88(1.77)	7(0.52)	33.12(0.41)	35.19	0.34
141	501(5.80)	245(3.54)	30.68(1.46)	42.13	0.45
143	226(3.27)	125(2.23)	29.98(0.80)	27.71	0.40
142	384(2.67)	297(6.41)	23.71(0.93)	22.34	0.49
147	635(8.99)	441(11.29)	20.15(1.67)	19.76	0.81
150	247(2.96)	204(2.93)	17.54(0.92)	16.52	0.37
167	650(8.40)	466(7.59)	16.84(1.75)	31.84	0.69
168	408(4.60)	340(4.96)	14.30(1.19)	40.36	0.52
181	262(6.61)	204(3.88)	3.05(1.27)	8.48	0.31
144	635(10.42)	592(6.56)	−1.38(1.51)	-4.36	0.54
149	502(10.44)	586(11.91)	−6.94(1.37)	21.72	0.58
166	437(8.54)	370(5.33)	−13.29(1.41)	-15.71	0.58

Table S3: Residue-level changes in the desolvation energy for nSH3:PRM$^{\mathrm{NS1}}$. See Table S2 for explanation.

Residue #	N^u_{cell}	N^b_{cell}	ΔG_{solv}	$\Delta G^{\mathrm{local}}_{\mathrm{solv}}$	C_α RMSF
190	403(8.05)	367(5.18)	28.15(1.53)	-9.23	2.92
188	264(3.69)	163(4.83)	25.49(1.47)	31.78	0.72
191	499(7.63)	458(5.00)	25.04(1.38)	-80.92	3.60
138	425(7.82)	398(9.51)	21.79(1.66)	20.22	0.55
135	407(7.13)	377(3.78)	16.81(1.80)	-3.57	0.96
165	495(6.21)	411(3.51)	9.44(1.49)	4.33	0.63
175	548(5.63)	504(2.23)	7.04(1.88)	2.66	0.80
182	10(0.46)	4(0.00)	6.81(0.15)	8.40	0.31
164	573(4.62)	507(7.60)	5.53(1.47)	5.00	0.53
170	228(4.60)	210(2.47)	5.21(1.04)	2.95	0.36
134	228(4.36)	429(5.71)	4.54(0.95)	-32.31	2.55
158	338(4.60)	357(4.57)	4.00(1.31)	13.40	0.54
140	342(4.88)	342(4.10)	3.61(1.28)	-2.24	0.52
187	84(2.62)	76(2.07)	3.32(0.46)	5.48	0.48
156	301(6.23)	303(2.87)	3.04(0.75)	2.25	0.55
155	568(8.12)	542(4.70)	0.48(1.41)	3.93	0.47
184	158(2.00)	170(2.59)	0.22(0.77)	9.55	0.45
163	447(5.04)	414(5.53)	−0.24(0.96)	-0.14	0.48
161	118(3.70)	118(2.51)	−0.99(0.62)	-0.35	0.43
159	68(1.58)	68(1.93)	−1.07(0.58)	2.26	0.43
139	40(1.77)	48(1.30)	−1.31(0.37)	0.63	0.45
177	347(3.93)	313(2.62)	−1.35(1.68)	-0.81	0.76
171	223(2.25)	212(5.18)	−1.85(1.02)	-4.17	0.34
151	68(1.85)	64(0.92)	−2.31(0.58)	-3.55	0.32
153	111(2.92)	109(2.00)	−2.82(0.51)	0.38	0.35
162	590(6.44)	599(7.49)	−3.74(1.84)	-1.38	0.44
180	94(1.51)	99(1.73)	−3.80(0.50)	-5.61	0.35
152	386(3.34)	376(3.81)	−3.82(1.35)	-3.42	0.39
157	253(3.11)	265(5.96)	−3.99(1.05)	0.95	0.55
173	240(3.23)	217(3.93)	−4.17(1.02)	-3.48	0.41
154	532(7.57)	538(4.72)	−4.38(1.87)	0.37	0.44
178	532(4.53)	507(7.60)	−5.06(1.91)	-19.28	0.63
160	399(7.09)	383(8.11)	−6.39(2.02)	-3.26	0.47
145	196(4.28)	175(3.62)	−6.80(0.88)	-1.72	0.62
137	20(0.92)	39(1.39)	−7.10(0.29)	-8.31	0.64
174	218(1.04)	241(3.85)	−9.94(0.78)	-2.84	0.54
179	623(8.57)	632(5.76)	−10.58(1.82)	-13.87	0.50
136	444(8.00)	392(2.90)	−14.54(1.40)	-21.03	0.80
176	754(6.92)	786(9.86)	−20.17(2.00)	-8.76	0.90
189	242(5.46)	389(6.14)	−25.35(1.09)	-7.68	1.75
148	717(12.70)	897(10.38)	−53.79(2.60)	-13.29	0.69
169	497(7.40)	112(3.65)	54.35(1.03)	59.04	0.41
143	226(3.27)	92(2.33)	40.05(0.92)	35.62	0.41
186	370(6.12)	87(1.60)	38.14(0.59)	39.39	0.45
181	262(6.61)	122(4.63)	36.30(1.39)	41.52	0.30
183	88(1.77)	6(0.35)	35.45(0.49)	37.18	0.37
150	247(2.96)	158(2.20)	34.96(1.09)	39.67	0.36
185	409(5.94)	293(2.83)	27.21(1.40)	36.01	0.54
142	384(2.67)	249(3.64)	24.59(0.85)	18.64	0.45
141	501(5.80)	335(3.30)	16.04(1.48)	26.74	0.47
166	437(8.54)	259(3.82)	2.00(1.34)	2.67	0.58
146	647(8.49)	532(6.14)	1.73(1.37)	-5.09	0.96
144	635(10.42)	535(4.63)	0.32(1.39)	4.20	0.55
149	502(10.44)	373(3.27)	−1.04(1.40)	54.53	0.54
172	3(0.83)	6(0.35)	−2.22(0.15)	-1.97	0.38
168	408(4.60)	422(5.34)	−7.33(1.40)	6.80	0.57
147	635(8.99)	421(6.80)	−13.94(1.71)	15.19	0.65
167	650(8.40)	650(7.32)	−19.46(2.00)	0.55	0.74

Table S4: Residue-level changes in the desolvation energy for nSH3:PRM^{cAbl} in simulations where protein atoms were positionally restrained with a 50-kcal/(mol·Å^2) harmonic potential. See Table S2 for explanation.

Residue #	N^u_{cell}	N^b_{cell}	ΔG_{solv}
176	626(2.17)	601(6.58)	8.66(1.88)
191	897(12.31)	879(8.25)	7.58(2.26)
175	426(0.46)	422(2.70)	6.85(1.06)
170	144(1.06)	144(2.31)	6.47(1.10)
179	559(1.55)	561(4.41)	6.29(2.64)
162	390(5.90)	391(5.28)	6.06(1.96)
190	576(6.93)	590(8.72)	4.44(2.98)
163	326(0.93)	337(2.36)	3.98(1.22)
134	361(2.33)	363(4.34)	3.86(1.51)
152	288(4.17)	287(3.63)	3.82(1.70)
158	224(3.46)	225(3.20)	3.71(1.82)
154	457(1.51)	472(3.18)	3.41(1.30)
151	38(2.12)	32(1.31)	3.41(0.40)
173	181(3.50)	167(2.45)	3.24(1.61)
165	303(4.53)	305(4.40)	3.13(1.60)
135	386(1.81)	383(4.10)	3.04(1.12)
136	487(9.50)	488(6.14)	2.82(1.20)
174	112(3.11)	112(0.71)	2.03(0.80)
180	75(0.64)	79(0.92)	1.85(0.66)
177	233(3.92)	227(5.23)	1.76(2.62)
145	167(3.24)	170(4.30)	1.41(1.32)
155	472(7.11)	454(5.45)	0.51(1.34)
171	163(3.62)	166(3.85)	0.37(0.89)
157	180(1.04)	183(2.00)	0.22(1.07)
164	381(3.00)	383(3.31)	0.03(1.51)
172	2(0.00)	2(0.00)	−0.04(0.03)
160	288(4.50)	308(4.56)	−0.56(2.42)
187	116(1.85)	109(1.51)	−0.74(0.61)
189	345(2.88)	366(4.14)	−0.98(3.30)
156	177(0.35)	181(2.76)	−1.08(0.77)
178	420(5.18)	421(4.17)	−1.18(2.16)
137	8(0.00)	9(0.35)	−2.12(0.18)
182	4(0.00)	9(0.74)	−3.06(0.22)
138	315(6.79)	321(3.07)	−4.45(1.39)
161	34(1.83)	40(1.51)	−4.94(0.53)
140	254(4.50)	245(3.85)	−7.57(2.14)
139	3(0.93)	11(0.71)	−8.39(0.15)
153	69(1.75)	76(1.19)	−9.33(0.59)
188	247(3.81)	269(4.17)	−9.39(1.59)
159	24(0.46)	34(0.52)	−10.16(0.41)
169	313(3.51)	49(0.46)	109.18(1.10)
141	490(2.19)	141(2.33)	87.16(1.94)
186	346(6.73)	56(1.51)	80.32(1.10)
185	353(4.84)	157(1.92)	58.80(0.94)
166	451(6.02)	304(2.75)	41.05(1.57)
183	44(1.55)	0(0.00)	29.44(0.11)
149	432(2.07)	411(4.96)	26.33(2.12)
147	383(4.75)	317(4.91)	26.10(2.03)
168	454(5.69)	370(6.90)	25.35(1.75)
146	585(9.61)	518(6.84)	20.71(1.85)
184	151(1.36)	129(1.60)	18.62(1.42)
150	109(3.50)	84(2.70)	18.17(1.19)
181	127(2.27)	110(2.19)	16.34(0.75)
143	118(0.93)	83(1.04)	16.05(1.32)
142	287(6.79)	266(4.21)	7.37(0.65)
148	709(14.98)	692(8.00)	7.11(2.70)
144	535(7.44)	530(5.72)	2.64(2.09)
167	599(6.13)	586(5.28)	−2.89(2.97)

Table S5: Residue-level changes in the desolvation energy for nSH3:PRM^{NS1} in simulations where protein atoms were positionally restrained with a 50-kcal/(mol·Å^2) harmonic potential. See Table S2 for explanation.

Residue #	N^u_{cell}	N^b_{cell}	ΔG_{solv}
160	285(4.50)	297(4.00)	6.38(2.05)
148	715(14.98)	711(7.11)	6.01(2.32)
164	417(3.00)	319(5.35)	3.42(1.88)
191	857(12.31)	856(8.96)	3.31(2.59)
184	162(1.36)	159(2.39)	3.12(1.43)
177	207(3.92)	213(4.29)	2.56(2.54)
162	473(5.90)	468(4.19)	2.10(1.92)
155	490(7.11)	492(6.79)	2.10(1.22)
175	448(0.46)	457(5.40)	1.57(1.03)
145	142(3.24)	133(3.11)	1.31(1.28)
156	205(0.35)	209(2.20)	1.21(0.91)
171	136(3.62)	147(2.33)	0.57(0.99)
159	22(0.46)	23(0.64)	0.36(0.29)
139	23(0.93)	23(1.41)	0.30(0.20)
182	0(0.00)	0(0.00)	0.00(0.00)
154	383(1.51)	383(4.06)	−0.40(1.37)
179	605(1.55)	617(5.96)	−0.47(2.97)
136	300(9.50)	314(6.08)	−0.70(1.32)
187	42(1.85)	40(1.36)	−0.78(0.71)
180	64(0.64)	61(1.06)	−0.83(0.39)
170	189(1.06)	194(1.96)	−1.15(1.44)
137	18(0.00)	18(0.00)	−1.18(0.14)
153	46(1.75)	46(0.35)	−1.49(0.41)
140	300(4.50)	295(3.52)	−1.67(1.88)
151	71(2.12)	76(1.30)	−1.79(0.59)
178	443(5.18)	462(3.02)	−1.92(2.07)
163	305(0.93)	313(4.02)	−2.63(1.06)
190	485(6.93)	498(4.34)	−2.82(3.02)
152	284(4.17)	304(4.99)	−3.01(1.67)
174	140(3.11)	155(2.83)	−3.17(0.76)
188	391(3.81)	402(4.28)	−3.88(1.41)
189	418(2.88)	441(3.46)	−4.59(2.53)
138	382(6.79)	429(2.82)	−12.00(1.06)
166	409(6.02)	85(2.00)	144.30(1.40)
169	314(3.51)	32(0.35)	121.34(1.06)
186	322(6.73)	50(0.93)	87.98(0.88)
165	383(4.53)	131(1.16)	63.54(1.17)
149	417(2.07)	294(3.55)	60.98(1.91)
141	491(2.19)	271(2.96)	53.46(2.16)
185	317(4.84)	176(1.55)	45.98(0.94)
181	148(2.27)	84(1.19)	37.58(0.63)
168	446(5.69)	332(6.00)	30.97(1.41)
147	391(4.75)	349(4.80)	28.62(1.84)
150	139(3.50)	95(2.13)	26.62(1.19)
142	298(6.79)	212(3.20)	24.42(0.77)
183	44(1.55)	0(0.00)	23.21(0.11)
143	96(0.93)	40(0.71)	20.44(1.17)
146	586(9.61)	497(7.33)	19.92(1.86)
167	580(6.13)	517(4.53)	18.36(2.60)
172	5(0.00)	5(0.00)	−1.01(0.07)
144	518(7.44)	472(4.98)	−4.23(2.02)
